# Pathogen characteristics are key determinants of distinct host response phenotypes of sepsis

**DOI:** 10.1172/JCI197346

**Published:** 2026-03-16

**Authors:** Rishi Chanderraj, Brian Bartek, Kathleen A. Stringer, Mohamad H. Tiba, Michael W. Sjoding, Ying He, Mark Nuppnau, Kale S. Bongers, Mark D. Adame, Sunny S. Lou, V. Eric Kerschberger, Matthew M. Churpek, Carolyn S. Calfee, Sandhya Tripathi, Debra M. Foster, John A. Kellum, Robert P. Dickson, Pratik Sinha

**Affiliations:** 1 Division of Infectious Diseases, Department of Internal Medicine, University of Michigan Medical School, Ann Arbor, Michigan, USA.; 2 Medicine Service, Infectious Diseases Section, Veterans Affairs (VA) Ann Arbor Healthcare System, Ann Arbor, Michigan, USA.; 3 Weil Institute for Critical Care Research & Innovation, Ann Arbor, Michigan, USA.; 4 Division of Clinical and Translational Research, Division of Critical Care, Department of Anesthesia, Washington University School of Medicine, St. Louis, Missouri, USA.; 5 Division of Pulmonary and Critical Care Medicine, Department of Internal Medicine, University of Michigan Medical School, Ann Arbor, Michigan, USA.; 6 Department of Clinical Pharmacy, College of Pharmacy,; 7 Department of Emergency Medicine,; 8 Institute for Healthcare Policy and Innovation, University of Michigan, Ann Arbor, Michigan, USA.; 9 Computational Medicine and Bioinformatics, University of Michigan Medical School, Ann Arbor, Michigan, USA.; 10 Division of Pulmonary, Critical Care and Occupational Medicine, Department of Internal Medicine, University of Iowa Medical School, Iowa City, Iowa, USA.; 11 Department of Microbiology and Immunology, University of Michigan Medical School, Ann Arbor, Michigan, USA.; 12 Department of Anesthesiology and; 13 Institute for Informatics, Data Science, and Biostatistics, Washington University School of Medicine, St Louis, Missouri, USA.; 14 Division of Allergy, Pulmonary, and Critical Care Medicine, Department of Medicine, Vanderbilt University Medical Center, Nashville, Tennessee, USA.; 15 Department of Medicine and; 16 Department of Biostatistics and Medical Informatics, University of Wisconsin–Madison, Madison, Wisconsin, USA.; 17 Division of Pulmonary, Critical Care, Allergy and Sleep Medicine, Department of Medicine,; 18 Department of Anesthesiology,; 19 Cardiovascular Research Institute, UCSF, San Francisco, California, USA.; 20 Spectral Medical Inc., Toronto, Ontario, Canada.; 21 Center for Critical Care Nephrology, Department of Critical Care Medicine, University of Pittsburgh, Pittsburgh, Pennsylvania, USA.

**Keywords:** Immunology, Infectious disease, Microbiology, Bacterial infections

## Abstract

**BACKGROUND:**

Sepsis encompasses considerable biological and clinical heterogeneity. Previously, 2 phenotypes (“hyperinflammatory” and “hypoinflammatory”) have been consistently identified within sepsis via latent class analysis. These phenotypes differ in their biological features, clinical outcomes, and therapeutic responses to interventions. Prior studies of sepsis heterogeneity have focused primarily on the host response. Here, we investigate the potential influence of the causative pathogen on sepsis heterogeneity and pathobiology.

**METHODS:**

We performed a retrospective observational analysis of 8,280 critically ill patients with sepsis to identify associations between pathogen characteristics and the hyperinflammatory and hypoinflammatory patient phenotypes. We also performed controlled murine and swine modeling of sepsis and lung injury and a secondary analysis of 449 patients enrolled in the EUPHRATES randomized controlled trial.

**RESULTS:**

Pathogen characteristics (pathogen identity, burden, virulence, and anatomic site of infection) were strongly and independently associated with the previously reported phenotypes. In a cohort of critically ill patients with sepsis, infection with gram-negative pathogens, primarily Enterobacterales spp. (e.g., *Escherichia coli*, *Klebsiella pneumoniae*), was strongly associated with the hyperinflammatory phenotype. The hyperinflammatory phenotype was also independently associated with increased pathogen burden, virulence, and initial anatomic site of infection. In controlled murine and swine modeling, both the identity and burden of the pathogen provoked key biological features of the hyperinflammatory phenotype. Among patients with sepsis, the prognostic value of lactate clearance varied substantially by phenotype. In a secondary analysis of a randomized trial of polymyxin B hemoadsorption (which removes circulating endotoxin), hypoinflammatory patients experienced worse survival.

**CONCLUSIONS:**

Our results demonstrate the central importance of pathogen features in the clinical and biological heterogeneity of sepsis. Future studies of sepsis pathobiology and heterogeneity should expand their scope beyond the host response, as understanding pathogen-host interactions will be crucial in the development of precision therapeutic strategies to improve patient outcomes.

**TRIAL REGISTRATION:**

EUPHRATES trial NCT01046669.

**FUNDING:**

5P30AG024824, IK2CX002766, R01HL144599, K24HL159247, R01HL158626, R01HL173531, R35GM142992, R35GM145330, R35GM136312, K23HL166880, R35HL140026.

## Abstract 2

Sepsis heterogeneity is shaped not only by host factors but also by pathogen identity, burden, and virulence, reframing our approach to precision therapies.

## Introduction

Sepsis, a syndrome of life-threatening organ dysfunction caused by infection (1), remains a major global health concern, accounting for an estimated 11 million deaths annually, or 20% of all deaths worldwide (2). Despite decades of research and numerous randomized clinical trials (3), mortality rates remain consistently above 20% (2, 4, 5). Due to its broad clinical definition (6), sepsis is a highly heterogeneous clinical diagnosis. The biological heterogeneity inherent in the clinical diagnosis of sepsis (7–9) has been implicated as a key factor leading to hundreds of negative trials evaluating targeted therapies (10, 11). As a result, there is a growing consensus that precision medicine approaches capable of identifying patient subgroups most likely to benefit from specific therapies are urgently needed (7–9).

While early antimicrobial therapy coupled with source control is the only intervention shown to improve sepsis mortality (12, 13), many patients still die, despite successful pathogen eradication (14). This paradox has led to the prevailing hypothesis that host factors, rather than the pathogen itself, are the primary determinants of sepsis biological and clinical heterogeneity (7, 15). This perspective, however, may have inadvertently overlooked the considerable role of specific pathogens and their interactions with the host in shaping the clinical course of sepsis (16). The potential for pathogen characteristics to contribute to the observed heterogeneity in sepsis has been largely unexplored.

Recent advances have identified 2 molecular phenotypes of sepsis — termed “hyperinflammatory” and “hypoinflammatory” — through latent class analysis, revealing distinct differences in their biological features, clinical outcomes, and therapeutic responses to randomized interventions (9, 17–20). In a recent study of 189 patients with sepsis using metagenomic sequencing, patients with the hyperinflammatory phenotype (*n* = 76) had a greater burden of bacterial DNA in the blood, enriched with members of the gram-negative *Enterobacterales* order, compared to hypoinflammatory patients.

Building on these insights, we aimed to determine the clinical significance of these findings in a real-world setting. Utilizing a validated machine learning model (9, 17), we identified phenotypes in a cohort of septic patients admitted to intensive care units at a tertiary hospital and examined the relationship between pathogens and sepsis phenotype. We recapitulated the key findings in murine and swine models and assessed the clinical implications of these findings in a secondary analysis of a prior randomized controlled trial of sepsis that evaluated the efficacy of an intervention that reduces circulating endotoxin. Our findings reveal that clinical markers of pathogen identity, burden, and virulence are independently associated with molecular phenotypes. To interrogate the clinical relevance of these findings, we demonstrate that in humans with sepsis, the prognostic utility of lactate clearance (a common resuscitation target in sepsis (21)), is modulated by both phenotype and causative pathogen, and we observed polymyxin B hemoadsorption was associated with worse survival among hypoinflammatory patients.

These findings challenge the prevailing host-centric paradigm of sepsis pathophysiology and suggest that integrating both host and pathogen factors, rather than focusing solely on host response, may be essential for identifying novel sepsis therapies. Future studies in sepsis should account for these important and understudied interactions.

## Results

### Sepsis phenotype assignment.

In a retrospective cohort of critically ill septic patients at the University of Michigan, we identified 8,280 patients who met our inclusion criteria (Supplemental Figure 1; supplemental material available online with this article; https://doi.org/10.1172/JCI197346DS1). The median probability of a hyperinflammatory phenotype in the cohort was 0.07, with a mean probability of 0.25 (Supplemental Figure 2). Using a probability threshold of 0.5 to classify patients into a binary hyper- or hypoinflammatory designation, 1,858 (22.4%) patients were classified as the hyperinflammatory phenotype and 6,422 (77.6%) as the hypoinflammatory phenotype (Table 1). To evaluate the temporal availability of the clinical data informing phenotype classification, we examined the timing of all laboratory and physiologic variables used as model inputs. Across 66,240 laboratory measurements, 65,898 (99.5%) represented the first obtained value, and 60,496 (91%) were drawn within 2 hours of presentation to the Emergency Department. The mean time to draw was 1.1 hours (median 0.43 hours; IQR 0.22–0.93 hours) from presentation. These results indicate that, although we used the operational framework of “worst-within-24-hours,” for input variable capturing, practically, the variables contributing to phenotype assignment overwhelmingly reflect initial presentation physiology.

### Validity of phenotype assignments.

To determine the validity of phenotype assignments, we asked if the clinical characteristics of phenotypes were similar to those of prior cohorts (9, 17, 19, 20) (Table 1). Concordant with previous studies, hyperinflammatory patients had higher acuity of illness (mean APACHE IV Score 52.5 hypoinflammatory, 83.0 hyperinflammatory, *P* < 0.001) and greater burden of comorbidities (mean Charlson comorbidity index 2.5 for hypoinflammatory; 3.4 for hyperinflammatory, *P* = 0.002) (Supplemental Table 1). Hyperinflammatory sepsis was also associated with more frequent bacteremia (23% in hypoinflammatory; 34% in hyperinflammatory, *P* < 0.001), greater duration of shock (mean vasopressor free–days 25.2 in hypoinflammatory; 18.3 in hyperinflammatory, *P* < 0.001), and higher 90-day mortality (21% for hypoinflammatory; 39% for hyperinflammatory, *P* < 0.001).

**Figure 1:**
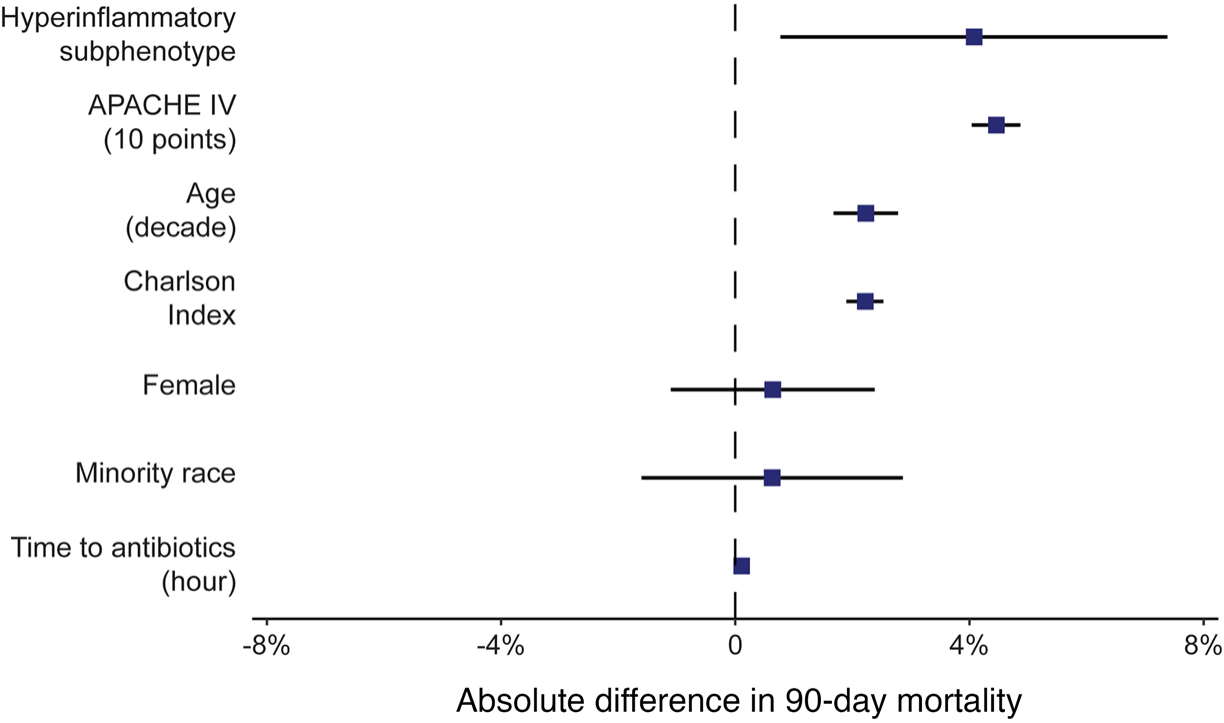
Hyperinflammatory subphenotype is independently predictive of 90-day mortality. In a cohort of 8,280 hospitalized patients with sepsis, classification within the hyperinflammatory subphenotype predicted increased 90-day mortality when controlled for severity of illness, comorbidities, demographics, and time to antibiotics.

We next asked if phenotype assignments identified clinically and prognostically meaningful information beyond disease severity and comorbid illness. To accomplish this, we built a multivariable logistic regression model of 90-day mortality, including phenotype assignment (as a binary hyper- or hypoinflammatory designation), demographics, APACHE IV score, Charlson comorbidity index, and time to antibiotic administration (to account for quality of sepsis care, given the strong association between time to antibiotic administration and sepsis mortality) as independent predictors in the model (Table 2 and Figure 1). We found that designation of the hyperinflammatory phenotype independently predicted 90-day mortality (OR 1.04, 95% CI 1.01–1.08, absolute marginal increase in ninety-day mortality 4.1%, 95% CI 0.8–7.4%). We concluded that phenotype assignment captured prognostically meaningful information, even when controlling for the severity of illness, age, and comorbidity burden. Results were consistent when 28-day mortality was used as the endpoint, with near-identical effect estimates for all covariates (hyperinflammatory phenotype OR 1.04, 95% CI 1.01–1.06, *P* = 0.0009) (Table 1 and Supplemental Table 2). Results were also similar when we modeled hyperinflammatory probability continuously on the 0–1 scale instead of as a binary label: higher probability remained independently associated with both 28- and 90-day mortality (Supplemental Table 3).

**Figure 2:**
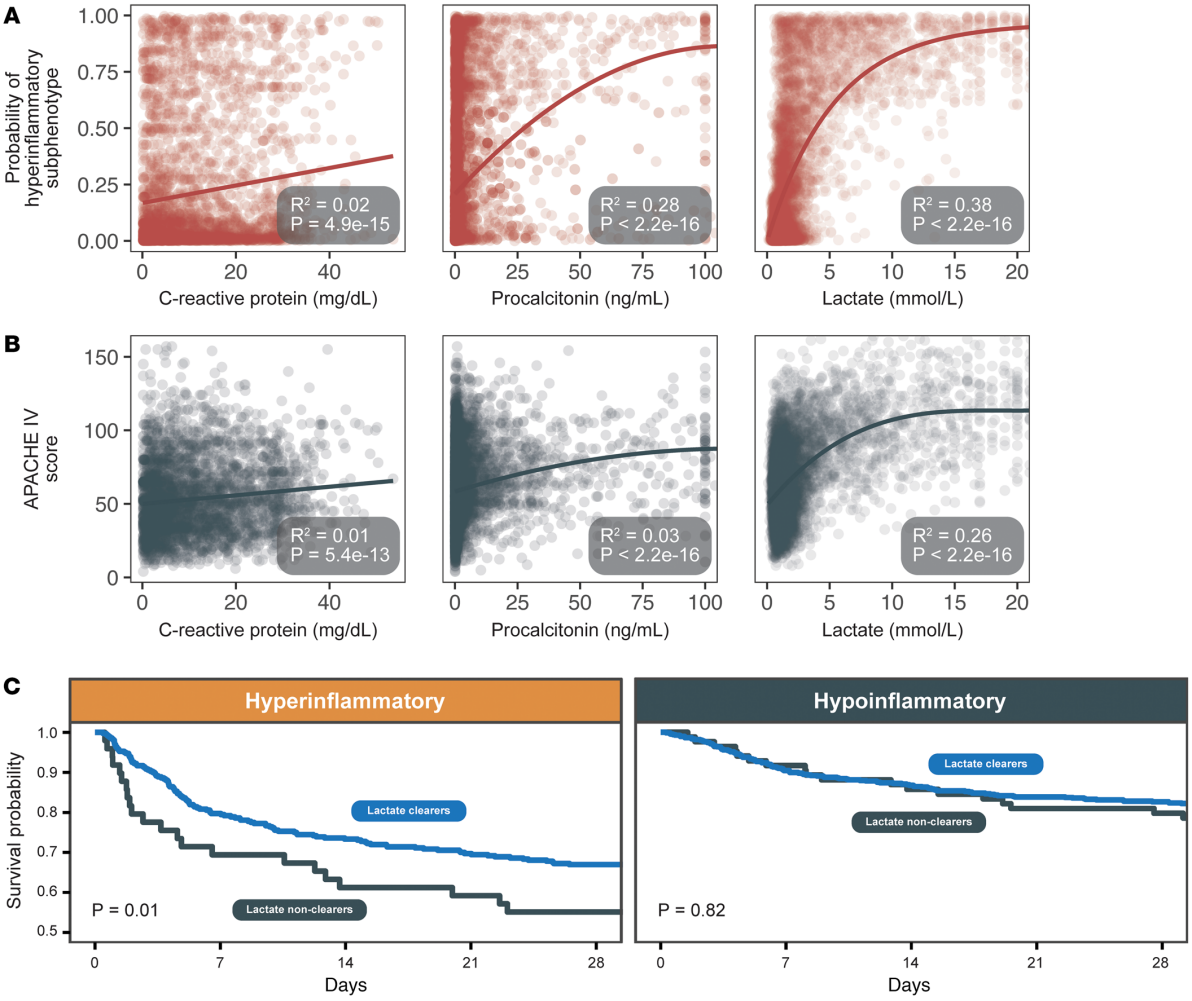
Hyperinflammatory subphenotype is associated with biomarkers of acute bacterial infection and shock. (**A**) Among 8,280 patients hospitalized with sepsis, the hyperinflammatory subphenotype was weakly correlated with C-reactive protein (a nonspecific inflammatory marker) and more strongly correlated with procalcitonin and lactate. (**B**) In contrast, physiologic severity of illness (APACHE IV) was only weakly associated both with C-reactive protein and procalcitonin. (**C**) In a cohort of 1,865 patients with vasopressor-dependent septic shock and elevated lactate, initial lactate clearance (defined as decreasing initial lactate concentration by 10% within 2–12 hours) was predictive of 28-day mortality in hyperinflammatory, but not hypoinflammatory, patients. This difference in predictive utility of lactate clearance was similar when comparing patients with and without Enterobacterales pathogens (Supplemental Figure 4).

We next asked whether these phenotypes capture biologically meaningful distinctions relevant to severe bacterial infection. To do this, we assessed 3 biomarkers, each representing a distinct facet of the host response. C-reactive protein (CRP) as a measure of general inflammation, procalcitonin as a measure of inflammation specific to bacterial infection (particularly gram-negative infections) (22), and lactate, though influenced by multiple factors, as a measure of hypoperfusion, mitochondrial dysfunction, and organ failure (1, 15, 21) (Figure 2, A and B). By examining these biologically grounded indices, we aimed to determine whether the hyperinflammatory phenotype aligns with a more exaggerated bacteria-driven inflammatory response and greater metabolic derangement than would be expected based on illness severity alone.

To distinguish phenotype-specific biology from global severity, we compared the fraction of variance (R^2^) in each biomarker explained by phenotype assignment versus APACHE IV. If these biomarkers primarily reflected global illness severity, their variance should be equally explained by APACHE IV and phenotype probability. Instead, among patients with available CRP measurements (*n* = 2,867), both APACHE IV and hyperinflammatory probability explained only about 1%–2% of the variance (R^2^ 0.014 versus R^2^ 0.017, respectively), reflecting weak overall associations. By contrast, among patients who had procalcitonin measured (*n* = 5,353), procalcitonin showed much stronger alignment with hyperinflammatory probability (R^2^ 0.28) than with APACHE IV (R^2^ 0.03). Among patients who had lactate measured (*n* = 7,964), lactate followed a similar pattern: phenotype probability explained roughly 38% of lactate’s variance, versus 26% for APACHE IV. Thus, while APACHE IV score captures biochemical perturbation associated with organ failure and nonspecific inflammation that is comparable to the hyperinflammatory phenotype, it fails to capture perturbations associated with bacteria-driven inflammation (procalcitonin), which is better captured by the hyperinflammatory phenotype. These findings demonstrate that the hyperinflammatory phenotype captures biological information beyond organ failure–derived severity scores.

### Clinical relevance of phenotype assignment: lactate clearance and survival.

Serum lactate and its clearance is a frequent target of hemodynamic resuscitation in sepsis (1, 15, 21), yet, despite several prospective trials (23, 24), its utility has never been demonstrated. We hypothesized that the additional 12% variance of lactate explained by the hyperinflammatory phenotype beyond the APACHE IV score may be due to the underlying pathophysiology of the phenotype. Specifically, we hypothesized that metabolic dysfunction, which has been observed in the hyperinflammatory phenotype, may explain this phenomenon (25), and that lactate clearance may be differentially prognostic according to the molecular phenotypes.

To test this hypothesis, we limited our analysis to a subgroup of patients who met inclusion criteria for the ANDROMEDA-SHOCK randomized clinical trial (23) (i.e., septic shock with vasopressor dependence and lactate ≥ 2.0 mmol/L, *n* = 1,865) in order to focus on a high-acuity population aligned with established resuscitation-focused research (23). We performed a multivariable Cox regression model of 90-day survival, adjusting for demographics, APACHE IV, Charlson comorbidity index, and time to antibiotic administration. We included an interaction term for lactate clearance × phenotype. This allowed us to determine whether the prognostic value of lactate clearance differs by phenotype, rather than assuming a uniform effect in all patients. In our multivariable analysis, we observed a significant interaction between lactate clearance and phenotype (HR 0.42, 95% CI 0.05–0.75). This estimate was well below 1.0, suggesting that early lactate reduction was associated with a stronger survival benefit in the hyperinflammatory phenotype than in the hypoinflammatory phenotype (Figure 2C and Supplemental Table 4). We thus concluded that the prognostic utility of lactate clearance is not uniform in sepsis but differs across phenotypes.

In our primary analysis, lactate clearance was defined as a ≥ 10% reduction in serum lactate within 6 hours of presentation, consistent with the 2021 Surviving Sepsis Campaign guidelines. To assess robustness, we repeated all analyses using the more stringent ANDROMEDA-SHOCK criterion of a ≥ 20% reduction every 2 hours. The direction and magnitude of associations, including the interaction between lactate clearance and phenotype, were materially unchanged (HR 0.42 [0.05–0.75] versus 0.13 [0.02–0.73]), confirming that findings were independent of the clearance definition (Supplemental Table 5).

### Association between pathogen features and phenotype.

We next sought to determine the association between patient pathogen features and phenotypes. We began our analysis by focusing on bacteremia. The most common causes of bacteremia in the overall cohort were *S*. *aureus* (*n* = 382) and *E*. *coli* (*n* = 215).

**Figure 3:**
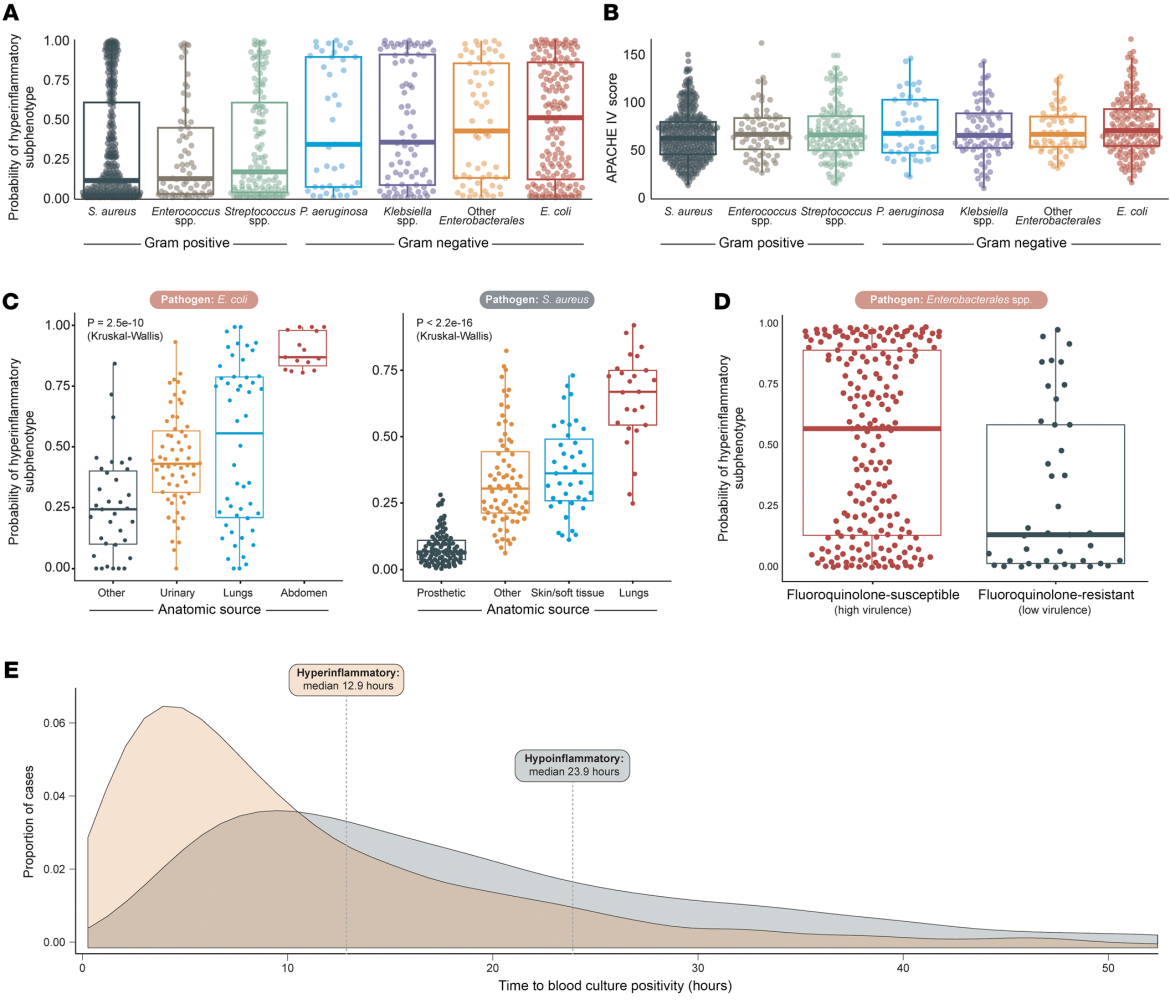
Hyperinflammatory subphenotype is predicted by pathogen features. (**A**) Among 2,108 bacteremic patients with sepsis, the hyperinflammatory subphenotype was strongly predicted by the identity of the pathogen, specifically gram-negative members of the Enterobacterales order (*E*. *coli*, *Klebsiella* spp.). (**B**) In contrast, physiologic severity of illness (APACHE IV) did not correlate strongly with pathogen identity. (**C**) Among patients with the same species of pathogen (*E*. *coli* [*n* = 161], *S*. *aureus* [*n* = 240]), the hyperinflammatory subphenotype was strongly predicted by anatomic site of initial infection. (**D**) Among patients infected with members of the Enterobacterales order (*n* = 248), in vitro resistance to fluoroquinolones (which is associated with decreased virulence) was predictive of the hypoinflammatory subphenotype. (**E**) Time to culture positivity, which is inversely correlated with blood bacterial burden, was shorter among patients in the hyperinflammatory subphenotype (*n* = 2,108).

To determine the association between pathogen identity and phenotype, we restricted our analysis to patients with monomicrobial bacteremia (*n* = 1,350). Pathogen identity was strongly associated with the likelihood of a hyperinflammatory phenotype (χ^2^ 106, df =11, *P* < 0.001, Supplemental Table 6 and Figure 3A). *E*. *coli* bacteremia had a higher probability of a hyperinflammatory phenotype than *S*. *aureus* (median probability 0.50 *E*. *coli*, 0.14 for *S*. *aureus,* *P* < 0.001). More broadly, patients with *Enterobacterales* spp. bacteremia were more likely to be classified as hyperinflammatory than patients with other pathogens (median probability 0.43 *Enterobacterales*, median probability 0.12 other pathogens, *P* < 0.001), and gram-negative organisms were more frequent among the hyperinflammatory phenotype (gram negative median probability 0.43, gram-positive median probability 0.12).

To determine whether these pathogen-phenotype associations observed in bacteremic patients extended to other infection types and to culture-independent pathogen detection, we conducted 2 complementary analyses. First, within our primary cohort we adjudicated infections in 891 nonbacteremic patients: 414 with lower respiratory infections (LRI) and 477 with urinary tract infections (UTI). Across both sites, gram-negative pathogens, particularly *Enterobacterales*, were strongly associated with the hyperinflammatory phenotype. Among patients with urinary infections, *Enterobacterales* infections showed higher hyperinflammatory probabilities than other pathogens (median 0.12 [IQR 0.02–0.58] versus 0.05 [0.01–0.35], *P* = 0.034). This pattern was mirrored in patients with LRI (median 0.10 [0.02–0.52] versus 0.09 [0.02–0.37], *P* = 0.0056). These findings parallel those observed in patients with bacteremia (Supplemental Figure 3 and Supplemental Table 7). Second, we analyzed patient data from the EUPHRATES trial, comparing circulating endotoxin concentrations (an index and mediator of gram-negative infection) with hyperinflammatory probability. Endotoxin activity correlated positively with hyperinflammatory probability across the full cohort (Spearman ρ = 0.13, *P* = 0.003) and within the hyperinflammatory subset (ρ = 0.21, *P* = 0.001) (Supplemental Figure 4). This relationship persisted despite approximately 70% of patients having negative blood cultures and the cohort being preselected for elevated endotoxin activity. Together, these complementary analyses demonstrate that the relationship between pathogen features and hyperinflammatory classification generalizes beyond bacteremia to other anatomic sites of infection in sepsis.

Given this strong relationship between *Enterobacterales* spp. bacteremia and the hyperinflammatory phenotype, we revisited our analysis of the prognostic utility of lactate clearance (Figure 2C), now asking whether the prognostic utility of lactate clearance varies according to pathogen identity. We again performed a multivariable Cox regression model, adjusting for demographics, APACHE IV, Charlson comorbidity index, and time to antibiotic administration (Supplemental Table 8). We now included an interaction term for lactate clearance × *Enterobacterales* infection to determine whether the prognostic value of lactate clearance differs by causative pathogen. As with phenotype status (Figure 2C), we observed a significant interaction between lactate clearance and *Enterobacterales* infection (HR 0.25, 95% CI 0.01–0.53) (Supplemental Figure 5). This estimate was also well below 1.0, suggesting that early lactate reduction was associated with a stronger survival benefit in patients with *Enterobacterales* spp. bacteremia.

We then asked whether the association between pathogen identity and hyperinflammatory phenotype was attributable to differences in sepsis severity. As shown in Figure 3B, sepsis severity (measured via APACHE IV score) did not correlate with pathogen identity. The association between pathogen identity and the hyperinflammatory phenotype (ω² = 0.02) was 2 orders of magnitude greater than the association between pathogen identity and APACHE IV score (ω² = 1 × 10^–4^), indicating that variation in illness severity (APACHE IV) is less associated with phenotype than pathogen identity. Another notable finding was that the distribution of hyperinflammatory probabilities among patients with gram-negative bacteremia was bimodal, with distributions polarized near 0 or 1, with lowest frequency of distribution at a probability of 0.5. This hourglass distribution of probabilities in gram-negative bacteremia contrasted both with patients with gram-positive bacteremia (in their phenotype probability, Figure 3A) and among all bacteremic patients in their APACHE IV scores (Figure 3B).

We then asked if the source (anatomic site) of bacteremia influenced the probability of a hyperinflammatory phenotype, focusing on the most common causes of bacteremia in the cohort: *E*. *coli* and *S*. *aureus* (Figure 3C and Supplemental Tables 9 and 10*)*. In patients with *E*. *coli* bacteremia, anatomic site of infection was associated with the likelihood of a hyperinflammatory phenotype (*P* < 0.001), with intraabdominal infection having the highest probability. The anatomic site of infection in *S*. *aureus* bacteremia was also associated with the likelihood of hyperinflammatory phenotype (*P* < 0.001), with pneumonia having the highest probability. We concluded that, alongside the identity of the pathogen, the anatomic source of bacteremia was also associated with the likelihood of hyperinflammatory phenotype.

We then characterized the association between virulence and phenotype by investigating the relationship between fluoroquinolone resistance and molecular phenotype in *Enterobacterales* bacteremia (Figure 3D), leveraging the established fitness cost associated with in vitro fluoroquinolone resistance in *Enterobacterales* (26–33). We found that fluoroquinolone resistance was associated with a decreased probability of hyperinflammatory phenotype (median probability 0.14 in resistant species, 0.57 in sensitive species, *P* < 0.001), suggesting an association between pathogen virulence and probability of hyperinflammatory phenotype.

Next, we characterized the association between phenotype and pathogen burden (Figure 3E). To accomplish this, we compared the time to positive blood culture growth between patients categorized as hyper- or hypoinflammatory, which is known to be inversely correlated with pathogen burden (34–38). When we dichotomized hyperinflammatory status at a probability threshold of 0.5, patients who were hyperinflammatory had a faster time to blood culture positivity compared with patients who were hypoinflammatory (mean time to positive 12.9 hours hyperinflammatory, 23.9 hours in hypoinflammatory, *P* < 0.001, Figure 3E). When evaluated as a continuous variable, the probability of a hyperinflammatory phenotype was negatively correlated with time to positive blood culture growth, with slower time to positivity (indicating lower bacterial burden) being significantly associated with a decreased likelihood of a hyperinflammatory phenotype (Spearman ρ= –0.22, *P* < 0.001, Supplemental Figure 6). These findings confirm our hypothesis that increased pathogen burden is positively correlated with hyperinflammatory phenotype status.

**Figure 4:**
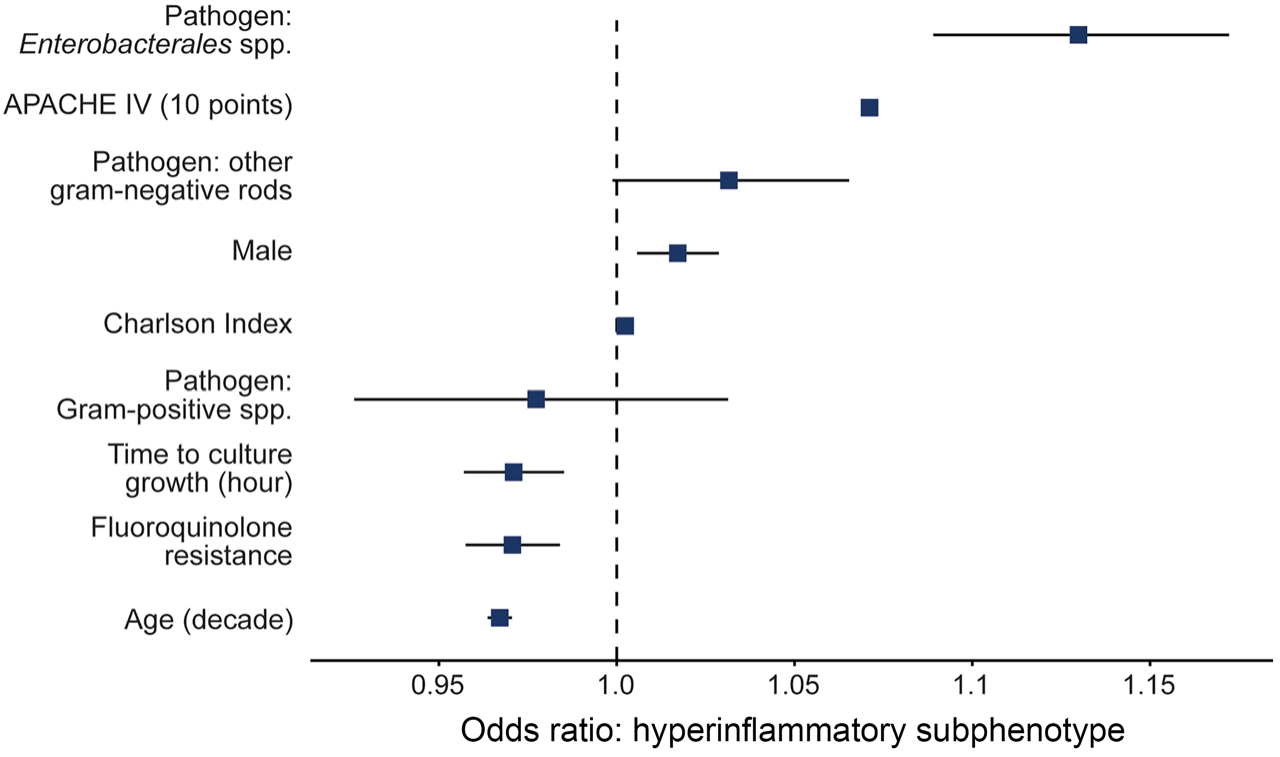
The identity, burden, and virulence of pathogens are independently predictive of hyperinflammatory subphenotype. Among 8,280 patients hospitalized with sepsis, bacteremia with a gram-negative pathogen, specifically *Enterobacterales* spp., was more predictive of hyperinflammatory subphenotype status than severity of illness, demographics, or comorbidities. Delayed bacterial culture growth (reflecting decreased bacterial burden) and fluoroquinolone resistance (reflecting decreased bacterial virulence) were negatively associated with hyperinflammatory status.

After determining that bacterial pathogens’ identity, burden, and virulence were linked to the likelihood of a hyperinflammatory phenotype, we developed a multivariable logistic regression model to assess the independent association of these characteristics with the phenotype (Figure 4 and Table 3). To achieve this, we included pathogen features that we identified as significant in the single variable analysis, as well as host features that we believed could play a role in the inflammatory response to a pathogen: the burden of medical comorbidities (characterized by Charlson index) and organ dysfunction (characterized by APACHE IV). Enterobacterales bacteremia was identified as the strongest independent predictor of the hyperinflammatory phenotype (OR 1.13, 95% CI 1.09 - 1.17). The time to blood culture growth and fluoroquinolone resistance were inversely correlated with burden and virulence, and both were also associated with the phenotype. Each hour of delay in blood culture growth correlated with reduced odds of a hyperinflammatory phenotype (OR 0.97, 95% CI 0.96–0.99), and fluoroquinolone resistance (OR 0.97, 95% CI 0.96–0.98), similarly decreased the odds of the hyperinflammatory phenotype.

### Swine and murine modeling.

We next set out to validate our observational human findings with complementary animal models. We started by using 2 swine models of acute lung injury/acute respiratory distress syndrome (ALI/ARDS), the first using only direct lung injury exposures (aspiration of clarified and acidified gastric particles, hyperoxia, and volutrauma) and the other using the same direct lung injury exposures as well as *E*. *coli* sepsis (as an indirect lung injury exposure). Details of the models, as well as physiologic and histopathologic confirmation of ALI/ARDS, have previously been published (39, 40). We analyzed 4 pigs in each experimental group and analyzed venous blood collected longitudinally during the experiment. We measured IL-6, IL-8, and bicarbonate to calculate the probability of hyperinflammatory phenotype (17).

**Figure 5:**
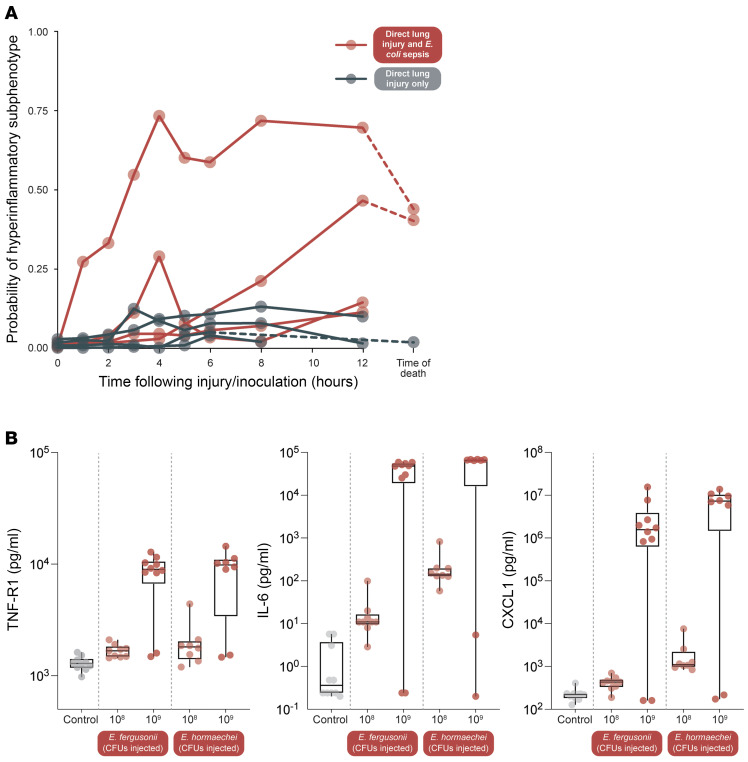
In animal models of ARDS and sepsis, hyperinflammatory biomarkers are provoked by *Enterobacterales* sepsis and increased pathogen burden. (**A**) In a swine model of ARDS, direct lung injury alone (aspiration, hyperoxia, volutrauma), did not provoke the biochemical features of the hyperinflammatory subphenotype, while combined direct lung injury and E. coli sepsis provoked the hyperinflammatory subphenotype in all exposed animals. (**B**) In a murine model of peritoneal sepsis, concentrations of blood biomarkers of the hyperinflammatory subphenotype (TNF-R1, IL-6, and CXCL1) were provoked by 2 models of Enterobacterales sepsis and potentiated by increased pathogen burden (increasing injected CFUs from 108 CFU to 109 CFU). Sepsis was modeled using intraperitoneal instillation of *E*. *fergusonii* and *E*. *hormaechei*. CXCL1 (also called KC) is the murine analogue to human IL-8.

The 4 animals who received only the direct lung injury exposures did not develop high probability of the hyperinflammatory phenotype (Figure 5A). In contrast, among the 4 animals who also had *E*. *coli* sepsis, the probability of classification as hyperinflammatory, while variable, was significantly higher (*P* < 0.05, comparison of peak probabilities). Biomarker-specific trajectories among animals are presented in Supplemental Figure 7. We thus concluded that the addition of *E*. *coli* sepsis to a model of ALI/ARDS provokes the biochemical and physiological features of hyperinflammatory sepsis/ARDS.

We then used murine modeling to test the hypothesis that, among animals with *Enterobacterales* sepsis, increased pathogen burden will provoke the biochemical features of hyperinflammatory sepsis. To accomplish this, we used a model of bacterial peritonitis that has previously been described (41) (Figure 5B). We used 2 different pathogens within the *Enterobacterales* order: *E*. *fergusonii* and *E*. *hormaechei*, and compared both with animals treated with saline controls. *Enterobacter hormaechei* strain ATCC 700323 was obtained from the American Type Culture Collection. The *E*. *fergusonii* strain was initially isolated from a mouse cecum and demonstrated to have high sequence homology to *Escherichia fergusonii* (a gift of Vincent Young, University of Michigan, Ann Arbor, Michigan, USA) (42). For each species, we compared 2 concentrations of inocula: 10^8^ and 10^9^ CFU. We measured blood concentrations of 3 key biomarkers of the hyperinflammatory phenotype: sTNF-R1, IL-6, and CXCL1 (the murine analogue for IL-8). For all 3 analytes, increased pathogen burden provoked a greater concentration of the hyperinflammatory biomarker (*P* < 0.05 for all comparisons). We thus concluded that, in a murine model of *Enterobacterales* sepsis, congruent with our human data (Figure 3E), development of the hyperinflammatory response is associated with increased pathogen burden.

### Clinical relevance of phenotype assignment: polymyxin B hemoadsorption.

Having established the relationship between gram-negative pathogens and the hyperinflammatory phenotype, we next asked whether the therapeutic benefit of polymyxin B hemoadsorption (which removes endotoxin from the bloodstream) varies by phenotype. We performed heterogeneity of treatment effect analysis of data from the EUPHRATES multicenter randomized controlled trial. Details regarding the trial and cohort have been published (43).

**Figure 6:**
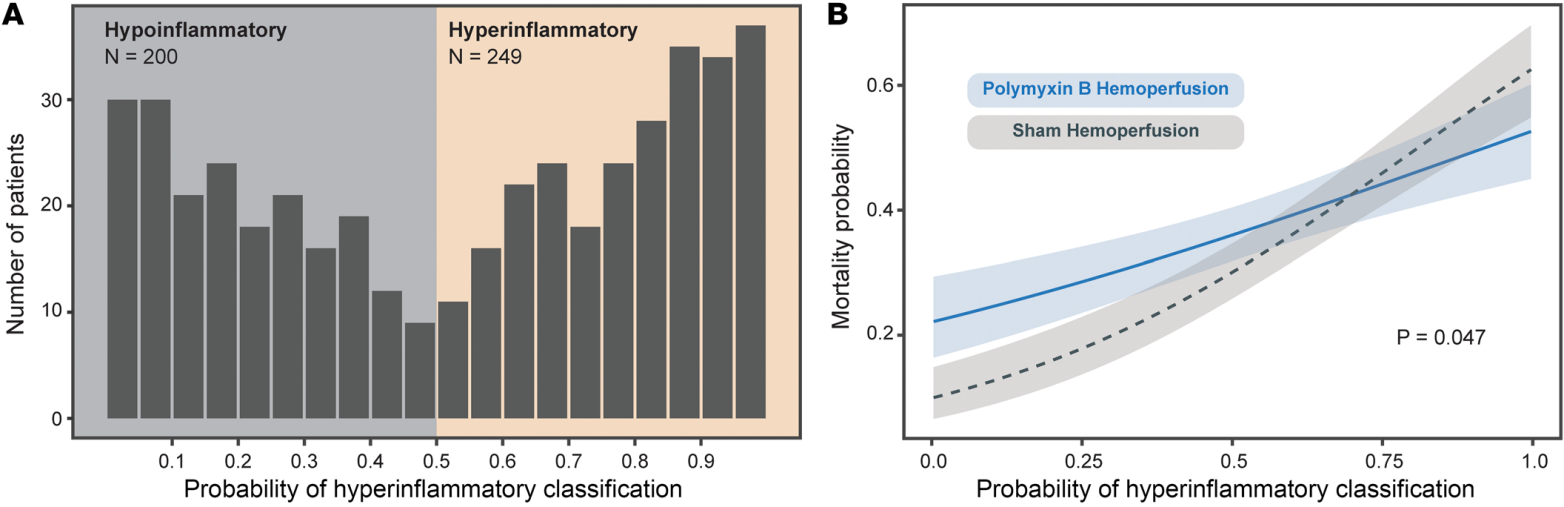
In sepsis, the therapeutic benefit of polymyxin B hemoperfusion depends on subphenotype status. (**A**) In the EUPHRATES clinical trial of polymyxin B hemoperfusion, the study population (for which endotoxemia was an inclusion criterion) was relatively enriched with the hyperinflammatory subphenotype. (**B**) We observed a significant interaction between hyperinflammatory subphenotype assignment probability and 28-day mortality.

Given that this cohort was enriched for elevated endotoxin activity, and given the observed relationship between gram negative pathogens and hyperinflammatory status (Figure 3A), we first hypothesized that hyperinflammatory patients would be enriched within the EUPHRATES cohort relative to other sepsis cohorts. As shown in Figure 6A, 45% (200 of 449) of patients were classified as hypoinflammatory and 55% (249 of 449) as hyperinflammatory. As predicted, this percentage of hyperinflammatory patients was greater than that of our primary cohort and that of prior published cohorts where the prevalence of the hyperinflammatory phenotype is approximately 30%–40% (17, 19, 20). Another notable finding in this endotoxin-enriched cohort was that distribution of probabilities was bimodal, recapitulating the findings in *Enterobacterales* bacteremic patients in the University of Michigan cohort.

We next hypothesized that the therapeutic benefit of endotoxin removal would be greater in the hyperinflammatory phenotype. We found a significant treatment interaction between randomization group and phenotype classification for the primary outcome of 28-day mortality (*P* = 0.041), with the polymyxin B hemoadsorption (treatment) arm associated with lower deaths in hyperinflammatory and higher deaths in hypoinflammatory patients compared with the sham group. This significant interaction was also observed when we used phenotype probability as a continuous variable (*P* = 0.047; Figure 6B). Notably, we found no significant treatment interaction between randomization group and severity of illness as measured using the MOD score (*P* = 0.395) or endotoxin activity levels (*P* = 0.417).

Although the interaction was statistically significant, the pattern of effect modification reflected lower survival with polymyxin B among patients at the hypoinflammatory end of the spectrum, with diminishing differences at higher hyperinflammatory probabilities. The curves converged beyond approximately 0.7, suggesting that endotoxin removal may be harmful in hypoinflammatory sepsis and, at best, neutral in hyperinflammatory sepsis. Accordingly, these findings are best interpreted as evidence that treatment efficacy varies by inflammatory state, with harm avoidance in hypoinflammatory sepsis emerging as the most robust inference.

## Discussion

Our study reveals that, in sepsis, numerous measurable features of the causative pathogen — identity, burden, virulence, and anatomic source — contribute to heterogeneity reflected in clinically important phenotypes. We found that pathogen identity, particularly infections with *Enterobacterales* (e.g., *E*. *coli* and *Klebsiella* spp.), is strongly associated with a hyperinflammatory phenotype, even after adjusting for traditional predictors of adverse outcomes in critical illness, such as the APACHE IV score and Charlson comorbidity index. Additionally, a shorter time to blood culture positivity — indicating a higher bacterial burden — was independently associated with the hyperinflammatory phenotype, while fluoroquinolone resistance, reflecting reduced bacterial virulence, was inversely related to this phenotype among *Enterobacterales* infections. Our swine and murine models provide experimental validation of these clinical observations: in a swine model of sepsis ALI/ARDS, *Enterobacterales* infection shifted the host response towards the hyperinflammatory phenotype, and in 2 murine models of *Enterobacterales* sepsis, increased pathogen burden provoked increased indices of the hyperinflammatory phenotype. Finally, the prognostic utility of lactate clearance and the therapeutic utility of polymyxin B hemoadsorption were both specific to patients in the hyperinflammatory phenotype. Taken together, these results suggest that pathogen characteristics are integral to the heterogeneity observed in sepsis, shaping the host response. The interaction of host with pathogen seems central to the development of the hyperinflammatory phenotype, which, in turn, determines pathophysiology, outcome, and, potentially, treatment response. These results reinforce the need to integrate pathogen-specific data more directly in future analyses of sepsis heterogeneity.

These findings underscore the critical influence of pathogen characteristics on the host response, complementing prior work that has emphasized intrinsic patient factors such as genetics and comorbidities. While our data link specific pathogens to distinct phenotypes, independent of general severity scores, they do not prove that microbial factors independently drive outcomes, nor do they negate additional sources of heterogeneity within the host response. Rather, they highlight the complex interplay between pathogen and host in sepsis pathobiology. Future investigations integrating genetic, environmental, and organism-specific data will be crucial for clarifying how these components collectively shape phenotypes and, ultimately, patient outcomes. From a clinical perspective, our findings indicate that both resuscitation targets (e.g., lactate clearance) and adjunctive treatments (e.g., endotoxin removal) may exert highly variable effects, depending on pathogen and phenotype. Lactate clearance, rather than serving as a universal prognostic marker, was particularly informative in patients with *Enterobacterales* infections and those exhibiting a hyperinflammatory state. Similarly, our secondary analysis of the EUPHRATES trial suggests that the benefit of endotoxin filtration is also context-specific. Collectively, these observations challenge a one-size-fits-all approach to sepsis management and underscore the importance of tailoring interventions to the biologic and microbiologic features of each patient.

While our study was not designed to resolve mechanism, our data directly suggest biological pathways that may be experimentally interrogated. Differences between gram-negative and gram-positive infections may reflect distinct microbial ligands and host receptors driving inflammation. Specifically, the observed correlation between endotoxin activity and hyperinflammatory probability provides molecular context for pathogen-driven host responses. Variation in endotoxin burden across *Enterobacterales* species, or in the structure of lipopolysaccharide (particularly the lipid A moiety) could modulate the potency of TLR4 engagement and amplify innate immune activation. Other pathogen-associated molecular patterns (e.g., flagellin, CpG DNA) may also contribute to differential activation, though endotoxin remains the most plausible driver. On the host side, genetic variation in TLR4 or its downstream adaptors (such as MyD88 and TRIF) may further influence responsiveness to endotoxin. Future integrative studies combining pathogen genomics, host receptor variation, and experimental models will be needed to test these causal links.

Our study is not without limitations. The retrospective design and reliance on EMR data introduce potential biases related to data quality and completeness. Although we employed rigorous statistical methods and adjusted for numerous confounders, the possibility of residual confounding remains. While our swine and murine models provide valuable experimental insights, individual models cannot fully capture the complexities and heterogeneity of human sepsis. Future studies should aim to validate these findings in diverse clinical settings and employ prospective designs to understand better the causal relationships between pathogen characteristics and sepsis phenotypes.

Our findings highlight several directions for future work. First, there is a need to define more precisely the molecular pathways through which pathogen features, such as burden and virulence, shape sepsis phenotypes. Such insights could guide discovery of novel therapeutic targets that modify the immune response in a pathogen- and phenotype-specific manner. Second, rather than treating pathogen identity and molecular phenotype as independent axes, future clinical trials might incorporate both pathogen-specific diagnostics and phenotype identification to refine enrollment criteria and therapeutic strategies. For instance, initiatives like the PANTHER trial consortium (http://panthertrial.org) are using an adaptive platform design to test phenotype-targeted therapies in ARDS, and analogous approaches could be extended to sepsis. Third, larger cohorts with detailed source adjudication are needed to determine whether specific pathogen-source combinations, such as gram negative intraabdominal infections, account for the bimodal distribution of hyperinflammatory probabilities we observed in gram-negative bacteremia. Finally, expanding this line of inquiry to a wider range of infections and pathogens (e.g., invasive fungal infections such as candidemia) will help confirm the generalizability of our findings and further advance the precision-medicine approach to sepsis.

In conclusion, our study highlights the importance of considering pathogen characteristics in understanding sepsis heterogeneity. By understanding both host and pathogen biology, we can move closer to achieving the goals of precision medicine in sepsis, ultimately improving outcomes for this complex and challenging syndrome.

## Methods

### Sex as a biological variable

Human cohorts included both male and female patients. Sex was included as a covariate in all multivariate models. In murine experiments, only female mice were used to minimize aggression-related variability in group housing. In swine experiments, only male animals were used. While the animal studies were not designed to assess sex-specific effects, the findings are interpreted within the context of established immunobiological mechanisms expected to be relevant across sexes.

### Study design and data source

We conducted a retrospective cohort study that analyzed Electronic Medical Record (EMR) data from the University of Michigan Research Data Warehouse.

### Population

Eligible patients were adults aged 18 and older who presented to the Emergency Department (ED) at the University of Michigan and were admitted to an intensive care unit (ICU) between July 1, 2016, and December 31, 2020. To identify patients with sepsis, we used the Centers for Disease Control (CDC) sepsis surveillance criteria, defined as (a) evidence of organ dysfunction, (b) blood cultures drawn at the time of presentation, (c) new initiation of antibiotic treatment lasting for at least 4 days (44, 45). We excluded patients who had previously been admitted for sepsis in the 90 days preceding the study start date and limited the analysis to the first admission for sepsis during the study period if patients had multiple sepsis admissions (Supplemental Figure 1).

### Identifying molecular phenotypes of sepsis

Hyperinflammatory and hypoinflammatory phenotypes in earlier studies were identified using latent class analysis (LCA) of plasma biomarkers and clinical data from observational cohorts and randomized clinical trials (9, 17). Notably, outcome and metrics of severity such as the APACHE score were not included as class-defining features in the LCA modelling. Because plasma biomarkers are not routinely available in clinical practice, in this cohort we used a previously developed and validated clinical-data-only model trained to classify these phenotypes (9, 17, 46). The model employs extreme gradient-boosting (XGBoost) and uses only data available in the EMR as inputs: temperature, heart rate, respiratory rate, systolic blood pressure, use of vasopressors, white blood cell count, sodium, glucose, creatinine, platelet count, bicarbonate and albumin.(9, 17) XGBoost is an optimized sequential decision tree model in which each successive tree refines the predictions of its predecessor. The final prediction is a weighted combination of all trees scaled by a learning rate (η) (46). Details of how these models were trained to predict the hyperinflammatory phenotype can be found in the original studies (9, 47) and the supplement. The model makes predictions of each patient’s classification and returns 2 outputs: the patient’s probability of belonging to the hyperinflammatory class and a binary indicator of the hyper- or hypoinflammatory class using a probability threshold 0.5 (48). This cut-off was predetermined during model derivation and validation in prior studies, calibrated to predict the original LCA-derived phenotype assignments. To align phenotype classification with the findings from culture specimens drawn at the time of hospital admission, we used the most abnormal clinical values from the first 24 hours after the Emergency Department presentation as inputs to classify patients into the phenotypes. Across 66,240 laboratory measurements used as input variables in the model, 65,898 (99.5%) represented the first obtained value during the index admission.

### Clinical data abstraction

Demographics, comorbidities, clinical laboratory values, vital signs, and medication administration data were abstracted from the University of Michigan Research Data Warehouse (RDW). We used the Charlson comorbidity index (49, 50) to characterize the burden of medical comorbidities in the cohort and the Acute Physiology and Chronic Health Evaluation IV (APACHE IV) Score (51) to quantify the severity of acute illness. For patient-centered outcomes, we used 90-day mortality as the primary outcome. 28-day mortality was used as a secondary outcome, given that it is a commonly used outcome in sepsis trials. Patient data were linked to the Social Security Death Index to identify postdischarge deaths (52).

### Pathogen classification

Given the high rate of misdiagnosis of infectious diseases (53–58), to study the association between pathogen identity and phenotypes, we focused on patients with a bacterial pathogen identified in admission blood cultures to minimize diagnostic uncertainty and identify patients with unambiguous bacterial infections. To maintain accuracy in pathogen identification, we excluded species commonly recognized as contaminants in blood cultures (59–61), specifically coagulase-negative *Staphylococcus* species other than *Staphylococcus lugdunensis* (62–65), diphtheroids without evidence of a concurrent prosthetic device infection (66), *Bacillus* species other than *Bacillus cereus,* and *Micrococcus* species (59–61).

Pathogens were classified to reflect their distinct roles in sepsis and their implications for clinical outcomes, with specific attention given to the most common pathogens identified by the Global Burden of Diseases Study (67). In single-variable analysis*, Escherichia coli* (*E*. *coli*), *Klebsiella* species, *Pseudomonas aeruginosa* (*P*. *aeruginosa*)*,* and *Staphylococcus aureus* (*S*. *aureus*) were categorized separately due to their high frequency, unique biological characteristics, and specific resistance patterns (67). *Streptococcus*, *Enterococcus*, and other Enterobacterales species were each categorized independently, given the tendency within each group to cause a similar spectrum of infections (67). Other pathogens were grouped based on their oxygen metabolism (strictly anaerobic or not) and gram-staining characteristics to facilitate the analysis of less common but clinically relevant infections.

To evaluate whether the findings in bacteremia replicated to pathogens isolated from other sites, we adjudicated nonbacteremic sterile-site infections, limiting our focus to lower respiratory tract and urinary tract infections (UTI), as they represent the most common nonbacteremic infection types that could be reliably classified at scale. Using the University of Michigan’s Electronic Medical Record Search Engine (EMERSE), we performed manual chart adjudication to confirm infection-related findings. Lower respiratory tract infection (LRI) required documentation of purulent sputum or respiratory secretions, new or worsening oxygen requirement, and radiographic evidence of a new infiltrate as previously described (68). UTI required compatible clinical features (e.g., dysuria, frequency, urgency, suprapubic pain, fever ≥ 38 °C, costovertebral tenderness, or hematuria) together with pyuria, absence of squamous epithelial contamination, and a urine culture ≥ 10^5^ CFU/mL of a single organism, as previously described (57).

When conducting multivariable analyses, pathogens were categorized into broader categories to minimize overfitting and reduce model complexity. Given prior metagenomic analyses highlighting their influence in the hyperinflammatory phenotype (20), *Enterobacterales* species were considered separately from other gram-negative and gram-positive organisms.

### Pathogen characteristics

To identify the anatomic source of bacteremia among patients infected with *E*. *coli* and *S*. *aureus*, we limited our analyses to patients with a microbiologic concordance between the culture taken from the anatomic source and the organism in the bloodstream. We then utilized a combination of approaches, including a review of available documentation in the EMR, claims-based algorithms, and imaging results (as described in prior studies (69)), to confirm that the growth of a pathogen in a sterile site culture represented an acute infection rather than chronic colonization.

We quantified bacterial burden in admission blood cultures by measuring the time to growth, defined as the interval from the sample’s arrival in the microbiology lab to the first detection of bacterial growth (34–38). The clinical microbiology lab at the University of Michigan monitors blood culture bottles containing nutrient broth with BD Bactec FX system (BD Diagnostics, Sparks, MD), which uses fluorescence-based sensors to detect changes in CO_2_ levels, signifying bacterial growth and metabolism. A shorter time to positivity indicates a higher bacterial burden (34–38). In in vitro studies, several bacterial species that develop fluoroquinolone (FQ) resistance often incur a measurable ‘fitness cost,’ which reduces their virulence (26–33). We hypothesized that this fitness cost would translate to a lower probability of hyperinflammatory phenotype. We used ciprofloxacin minimum inhibitory concentration (MIC) values reported by the University of Michigan clinical microbiology laboratory to define resistance. An isolate was classified as resistant if the minimal inhibitory concentration for ciprofloxacin was equal to or greater than 1 μg/mL, consistent with Clinical and Laboratory Standards Institute (CLSI) standards (70).

### Serum lactate analysis

To test whether responses to resuscitation strategies varied by phenotype and by a causative pathogen in the University of Michigan cohort, we performed a subgroup analysis in a population of patients that met the inclusion criteria for the ANDROMEDA-SHOCK randomized clinical trial (23). This trial was designed to test the efficacy of resuscitation guided by serum lactate clearance compared with resuscitation guided by peripheral perfusion and showed no difference in outcomes between the 2 groups. Thus, patients in this subgroup analysis (a) had an elevated serum lactate level (> 2.0 mmol/L) drawn within 24 hours of presentation to the Emergency Department and (b) required vasopressors to maintain a mean arterial pressure of 65 mm Hg or higher. Patients excluded from this subgroup analysis (a) had do-not-resuscitate status, (b) were admitted for bleeding from any source, or (c) were admitted for acute respiratory distress syndrome. As a sensitivity analysis, we further limited this subgroup to patients with severe lactate elevation, defined as a starting serum lactate of > 4.0 mmol/L, which we believed to be a more clinically pertinent level, and defined lactate clearance using the 20% reduction criterion employed in the ANDROMEDA-SHOCK trial.

### Heterogeneity of treatment effect: randomized trial of polymyxin B hemoadsorption (EUPHRATES)

This analysis was a secondary analysis of the EUPHRATES trial, which tested the efficacy of polymyxin B versus sham hemoadsorption among critically ill patients with septic shock, and its details can be found in the original manuscript (43). Briefly, the trial enrolled 450 adult patients with septic shock and bloodstream endotoxin activity levels of 0.60 or higher across 55 tertiary hospitals in North America between September 2010 and June 2016. One patient was excluded due to the unavailability of primary outcome data. Endotoxin levels were quantified using the Endotoxin Activity Assay (EAA) (Spectral Medical, Toronto, Canada) (71). Patients were randomized 1:1 to either polymyxin B hemoadsorption, with a cartridge designed to bind and remove endotoxin from the bloodstream (72), or sham hemoadsorption. Each patient received 2 hemoadsorption treatments within 24 hours with a target treatment duration of 2 hours each.

The original trial showed no significant treatment benefit, including in prespecified subgroup analyses. We analyzed anonymized patient data on all enrolled patients in the EUPHRATES trial, including information on treatment allocation, clinical and microbiological characteristics at study enrollment, and outcomes.

Sepsis phenotypes were identified using the same validated clinical data–only classifier model described above for the primary cohort (9, 17, 46). For this analysis, prerandomization patient data were used as input features for the model.

### Swine and murine modeling

#### Swine.

Direct lung injury and combined direct/indirect lung injury was modeled in 8 Yorkshire swine, as previously described (39, 40) (4 animals in each experimental group). Briefly, direct lung injury was provoked using hyperoxia (FiO_2_ 100%), volutrauma (Vt 15 ml/kg), and aspiration of clarified and acidified gastric particles. Indirect lung injury was provoked using *E*. *coli* sepsis via direct inoculation of live *E*. *coli* (average culture count of 2.5 × 10^11^ CFUs) into the kidney parenchyma. Blood was collected from the internal jugular vein at every hour for the first 6 hours, then every 2 hours until hour 12 and again at the time of death. To classify pigs into the hyperinflammatory phenotype, we developed a 3 variable logistic regression classifier model using IL-6, IL-8, and serum bicarbonate, using methodologies previously described (17). This logistic regression model was then used to generate probabilities of belonging to the hyperinflammatory phenotype at each timepoint.

#### Murine.

Modeling of *Enterobacterales* sepsis was performed using peritoneal instillation of 2 bacterial species: *E*. *fergusonii* and *E*. *hormaechei* (both members of the *Enterobacterales* order). Experimental details have previously been published (41). Briefly, bacteria were injected into the peritoneum at reported inoculum concentrations, and mice were harvested 24 hours later. Blood was obtained by puncture of the right ventricle with a heparinized syringe, and plasma was prepared by centrifugation at 2,000*g* for 15 minutes. Analytes were measured via Luminex assay, as previously reported (41, 73).

### Statistics

Associations between binary molecular phenotype classifications (hyper- and hypoinflammatory) and clinical variables were evaluated using χ² tests for categorical variables and 2-tailed *t* tests for continuous variables. We used Spearman correlation coefficients to quantify the relationships between plasma biomarkers used to measure inflammation and organ dysfunction and the probability of a hyperinflammatory phenotype and the APACHE IV Score, respectively: C-reactive protein (as a marker of generalized inflammation), procalcitonin (as a marker of inflammation specific to bacterial infection), and serum lactate (as a marker of organ dysfunction). For these analyses that compared the fraction of phenotype or APACHE IV variance (R²) explained by the biomarkers, only available measurements were used; no imputation or inference was performed for missing data. Biomarker availability varied across the cohort: C-reactive protein (CRP) was measured in 2,867 individuals (34.6%), procalcitonin in 5,353 individuals (64.7%), and lactate in 7,964 individuals (96.2%). Because these analyses were designed to assess relative explanatory strength rather than population-level biomarker distributions, restricting to observed data avoided introducing assumptions about the mechanism of missingness and preserved analytic integrity. We used the Kruskal-Wallis test to determine if the causative pathogen was associated with the likelihood of a hyperinflammatory phenotype or APACHE IV. The Wilcoxon rank-sum test was used to compare the probability of a hyperinflammatory phenotype between specific pathogen groups. Survival differences across binary phenotypes and pathogen categories were compared using the log-rank test.

To compare the strength of the association between pathogen identity and molecular phenotypes versus severity of illness, we constructed linear regression models for each outcome as a function of pathogen identity. We quantified the strength of each association using omega squared (ω²), allowing for a direct comparison of the variance explained while accounting for differences in scale.

For multivariable analysis, we fit logistic regression models to predict phenotypes based on clinical variables not included in the original latent class analysis (LCA) derivation, including pathogen characteristics (identity, time to growth, and fluoroquinolone resistance), Charlson Comorbidity Index, and APACHE IV Score. We used a 10-point increase in APACHE IV score in these models to model a clinically meaningful change in illness severity. We also used logistic regression to determine the independent association between molecular phenotype and 90-day mortality; covariates included demographics, Charlson Comorbidity Index, time to antibiotic administration, and APACHE IV Score. In additional sensitivity analyses, we repeated the mortality models using the continuous predicted probability of hyperinflammatory classification (0–1 scale) rather than a binary hyperinflammatory/hypoinflammatory designation. We fit multivariable logistic regression models for 28 day and 90 day mortality, including the continuous hyperinflammatory probability, age (per decade), Charlson comorbidity index, APACHE IV score, sex, racial minority status, and time to antibiotic administration as predictors. Marginal effects were calculated to determine absolute changes in 90-day mortality. We performed a multivariable Cox regression model of 90-day survival, adjusting for demographics, APACHE IV, Charlson comorbidity index, and time to antibiotic administration. We included an interaction term for lactate clearance × phenotype.

To test for treatment interaction between randomized treatment group allocation and molecular phenotype, we built a logistic regression model with 28-day mortality as the dependent variable and treatment group, phenotype assignment, and their interaction term as the independent variables. A 2-sided *P* value of < 0.05 for the interaction term was considered statistically significant heterogeneous treatment effect. To evaluate whether there might be a nonlinear interaction between hyperinflammatory phenotype assignment and treatment group, we repeated the analyses using the continuous probability of hyperinflammatory phenotype assignment generated by the clinical classifier model instead of the dichotomous phenotype classification.

All analyses were performed in R version 4.3.2.

### Study approval

The University of Michigan Institutional Review Board deemed the study exempt from needing consent under the United States federal regulations to protect human research subjects (45 CFR §46, category 4, secondary use of identifiable data). This study followed the Strengthening the Reporting of Observational Studies in Epidemiology (STROBE) reporting guideline.

All animal studies were approved by the Institutional Animal Care and Use Committee at the University of Michigan (PRO00011460 and PRO00012061). Laboratory animal care policies at the University of Michigan follow the Public Health Service Policy on Humane Care and Use of Laboratory Animals.

### Data availability

The University of Michigan Institutional Review Board deemed this study exempt from needing consent under the United States federal regulations to protect human research subjects (45 CFR §46, category 4, secondary use of identifiable data). Patient-level data will not be shared publicly to secure patient identities and protected health information. Aggregate data is provided in the Supplement, and additional aggregate data will be provided upon request. Values for all data points in graphs are reported in the Supporting Data Values file.

## Author contributions

RC, RPD, and PS conceived the study and designed the analyses. RC, BB, MWS, YH, MN, MDA, JAK, DMF, SSL, VEK, and PS obtained, curated, and analyzed the human clinical data. KAS, MHT, and RPD performed the swine experiments and generated the relevant data. KSB and MDA performed the murine experiments and generated the relevant data. RC, BB, KAS, MWS, YH, MN, MDA, KSB, ST, RPD, MMC, CSC, and PS participated in data analysis and interpretation. RC wrote the original draft of the manuscript. All authors had access to the study data, contributed to data interpretation, critically reviewed the manuscript, and approved the final version. RPD and PS contributed equally to this study.

## Funding support

This work is the result of NIH funding, in whole or in part, and is subject to the NIH Public Access Policy. Through acceptance of this federal funding, the NIH has been given a right to make the work publicly available in PubMed Central.

- 5P30AG024824 and IK2CX002766 (RC).
- R01HL144599, K24HL159247 (RPD).
- R01HL158626 (MS).
- R01HL173531, R35GM142992 (PS).
- K08AR083015 (KSB).
- R35GM145330 (MC).
- R35GM136312 (KAS).
- K23HL166880 (SL).
- R35HL140026 (CSC).
- The Ann Arbor VA Medical Center.

## Supplementary Material

Supplemental data

ICMJE disclosure forms

Supporting data values

## Figures and Tables

**Table 1:**
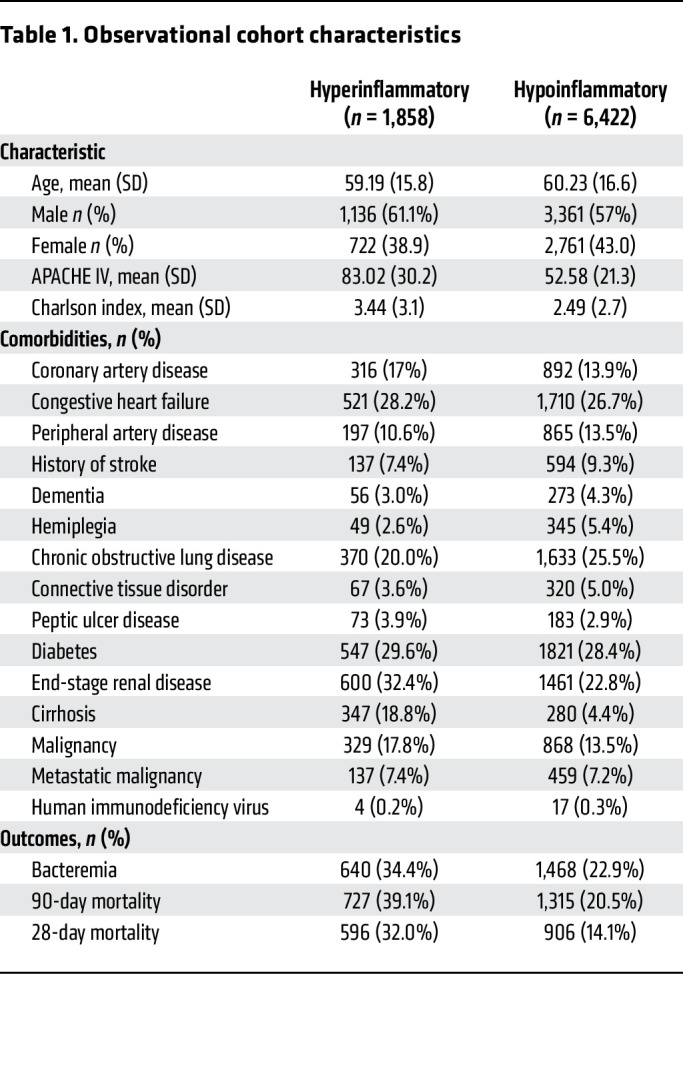
Observational cohort characteristics

**Table 3:**
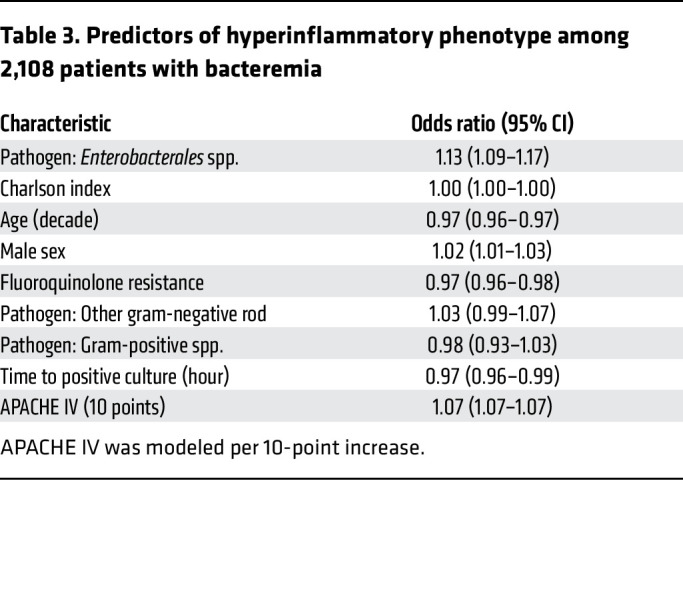
Predictors of hyperinflammatory phenotype among 2,108 patients with bacteremia

**Table 2:**
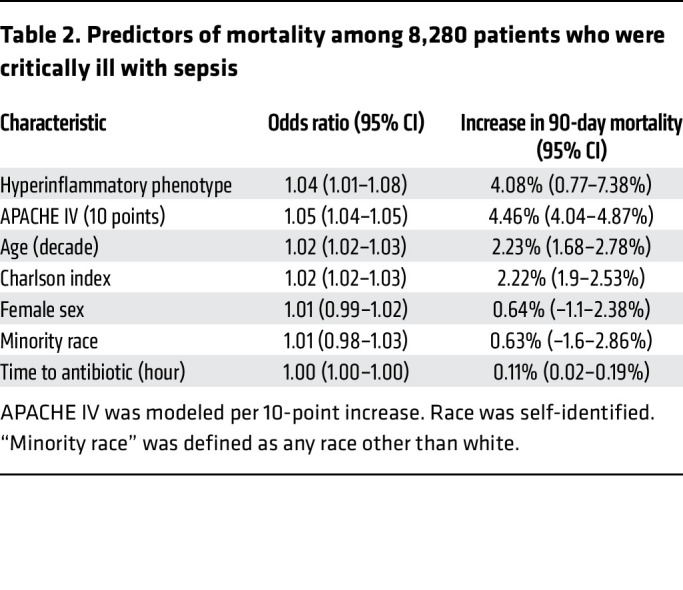
Predictors of mortality among 8,280 patients who were critically ill with sepsis

## Version 1 03/16/2026

Electronic publication

## References

[1] Singer M (2016). The third international consensus definitions for sepsis and septic shock (Sepsis-3). JAMA.

[2] Rudd KE (2020). Global, regional, and national sepsis incidence and mortality, 1990-2017: analysis for the Global Burden of Disease Study. Lancet.

[3] Cavaillon JM (2020). Sepsis therapies: learning from 30 years of failure of translational research to propose new leads. EMBO Mol Med.

[4] Stevenson EK (2014). Two decades of mortality trends among patients with severe sepsis: a comparative meta-analysis*. Crit Care Med.

[5] Bauer M (2020). Mortality in sepsis and septic shock in Europe, North America and Australia between 2009 and 2019- results from a systematic review and meta-analysis. Crit Care.

[6] Seymour CW (2016). Assessment of clinical criteria for sepsis: for the third international consensus definitions for sepsis and septic shock (Sepsis-3). JAMA.

[7] Shankar-Hari M (2024). Reframing sepsis immunobiology for translation: towards informative subtyping and targeted immunomodulatory therapies. Lancet Respir Med.

[8] Leligdowicz A, Matthay MA (2019). Heterogeneity in sepsis: new biological evidence with clinical applications. Crit Care.

[9] Sinha P (2023). Identifying molecular phenotypes in sepsis: an analysis of two prospective observational cohorts and secondary analysis of two randomised controlled trials. Lancet Respir Med.

[10] Vincent JL (2022). The end of “one size fits all” sepsis therapies: toward an individualized approach. Biomedicines.

[11] Marshall JC (2014). Why have clinical trials in sepsis failed?. Trends Mol Med.

[12] Liu VX (2017). The timing of early antibiotics and hospital mortality in sepsis. Am J Respir Crit Care Med.

[13] Seymour CW (2017). Time to treatment and mortality during mandated emergency care for sepsis. N Engl J Med.

[14] Thaden JT (2022). Association of follow-up blood cultures with mortality in patients with gram-negative bloodstream infections: a systematic review and meta-analysis. JAMA Netw Open.

[15] Angus DC, van der Poll T (2013). Severe sepsis and septic shock. N Engl J Med.

[16] Casadevall A, Pirofski LA (2000). Host-pathogen interactions: basic concepts of microbial commensalism, colonization, infection, and disease. Infect Immun.

[17] Sinha P (2020). Development and validation of parsimonious algorithms to classify acute respiratory distress syndrome phenotypes: a secondary analysis of randomised controlled trials. Lancet Respir Med.

[18] Heijnen NFL (2021). Biological subphenotypes of acute respiratory distress syndrome show prognostic enrichment in mechanically ventilated patients without acute respiratory distress syndrome. Am J Respir Crit Care Med.

[19] Calfee CS (2018). Acute respiratory distress syndrome subphenotypes and differential response to simvastatin: secondary analysis of a randomised controlled trial. Lancet Respir Med.

[20] Neyton LPA (2024). Host and microbe blood metagenomics reveals key pathways characterizing critical illness phenotypes. Am J Respir Crit Care Med.

[21] Evans L (2021). Surviving sepsis campaign: international guidelines for management of sepsis and septic shock 2021. Intensive Care Med.

[22] Simon L (2004). Serum procalcitonin and C-reactive protein levels as markers of bacterial infection: a systematic review and meta-analysis. Clin Infect Dis.

[23] Hernández G (2019). Effect of a resuscitation strategy targeting peripheral perfusion status vs serum lactate levels on 28-day mortality among patients with septic shock: The ANDROMEDA-SHOCK randomized clinical trial. JAMA.

[24] Jones AE (2010). Lactate clearance vs central venous oxygen saturation as goals of early sepsis therapy: a randomized clinical trial. JAMA.

[25] Alipanah-Lechner N (2023). Plasma metabolic profiling implicates dysregulated lipid metabolism and glycolytic shift in hyperinflammatory ARDS. Am J Physiol Lung Cell Mol Physiol.

[26] Yang S (2006). Quorum sensing and multidrug transporters in Escherichia coli. Proc Natl Acad Sci U S A.

[27] Praski Alzrigat L (2021). Resistance/fitness trade-off is a barrier to the evolution of MarR inactivation mutants in Escherichia coli. J Antimicrob Chemother.

[28] Horváth A (2012). Varying fitness cost associated with resistance to fluoroquinolones governs clonal dynamic of methicillin-resistant Staphylococcus aureus. Eur J Clin Microbiol Infect Dis.

[29] Tóth A (2014). Fitness cost associated with resistance to fluoroquinolones is diverse across clones of Klebsiella pneumoniae and may select for CTX-M-15 type extended-spectrum β-lactamase. Eur J Clin Microbiol Infect Dis.

[30] Rozen DE (2007). Fitness costs of fluoroquinolone resistance in Streptococcus pneumoniae. Antimicrob Agents Chemother.

[31] Melnyk AH (2015). The fitness costs of antibiotic resistance mutations. Evol Appl.

[32] Johnson CN (2005). Relative fitness of fluoroquinolone-resistant Streptococcus pneumoniae. Emerg Infect Dis.

[33] Wang YP (2009). Quinolone-resistance in Salmonella is associated with decreased mRNA expression of virulence genes invA and avrA, growth and intracellular invasion and survival. Vet Microbiol.

[34] Chen Y (2020). Prognostic roles of time to positivity of blood cultures in patients with *Escherichia coli* bacteremia. Epidemiol Infect.

[35] Khatib R (2005). Time to positivity in Staphylococcus aureus bacteremia: possible correlation with the source and outcome of infection. Clin Infect Dis.

[36] Marra AR (2006). Time to blood culture positivity as a predictor of clinical outcome of Staphylococcus aureus bloodstream infection. J Clin Microbiol.

[37] Opota O (2015). Blood culture-based diagnosis of bacteraemia: state of the art. Clin Microbiol Infect.

[38] Hsu MS (2014). Sequential time to positivity of blood cultures can be a predictor of prognosis of patients with persistent Staphylococcus aureus bacteraemia. Clin Microbiol Infect.

[39] Tiba MH (2020). A comprehensive assessment of multi-system responses to a renal inoculation of uropathogenic E. coli in swine. PLoS One.

[40] Tiba MH (2021). A novel swine model of the acute respiratory distress syndrome using clinically relevant injury exposures. Physiol Rep.

[41] Bongers KS (2024). Inflammatory responses to polymicrobial intra-abdominal sepsis are highly variable but strongly correlated to enterobacteriaceae outgrowth. Shock.

[42] Bongers KS (2025). Gut microbiome-mediated nutrients alter opportunistic bacterial growth in peritonitis. Am J Physiol Gastrointest Liver Physiol.

[43] Dellinger RP (2018). Effect of targeted polymyxin b hemoperfusion on 28-day mortality in patients with septic shock and elevated endotoxin level: the EUPHRATES Randomized Clinical Trial. JAMA.

[44] Rhee C (2017). Incidence and trends of sepsis in US hospitals using clinical vs claims data, 2009-2014. JAMA.

[47] Sinha P (2020). Machine learning classifier models can identify acute respiratory distress syndrome phenotypes using readily available clinical data. Am J Respir Crit Care Med.

[48] Maddali MV (2022). Validation and utility of ARDS subphenotypes identified by machine-learning models using clinical data: an observational, multicohort, retrospective analysis. Lancet Respir Med.

[49] Charlson ME (1987). A new method of classifying prognostic comorbidity in longitudinal studies: development and validation. J Chronic Dis.

[50] Quan H (2011). Updating and validating the Charlson comorbidity index and score for risk adjustment in hospital discharge abstracts using data from 6 countries. Am J Epidemiol.

[51] Zimmerman JE (2006). Acute Physiology and Chronic Health Evaluation (APACHE) IV: hospital mortality assessment for today’s critically ill patients. Crit Care Med.

[52] Schisterman EF, Whitcomb BW (2004). Use of the Social Security Administration Death Master File for ascertainment of mortality status. Popul Health Metr.

[53] Jones BE (2024). Diagnostic discordance, uncertainty, and treatment ambiguity in community-acquired pneumonia: a national cohort study of 115 U.S. Veterans Affairs Hospitals. Ann Intern Med.

[54] Gupta AB (2024). Inappropriate diagnosis of pneumonia among hospitalized adults. JAMA Intern Med.

[55] Cutler TS (2023). Prevalence of misdiagnosis of cellulitis: A systematic review and meta-analysis. J Hosp Med.

[56] Daniel P (2017). Adults miscoded and misdiagnosed as having pneumonia: results from the British Thoracic Society pneumonia audit. Thorax.

[57] Petty LA (2019). Risk factors and outcomes associated with treatment of asymptomatic bacteriuria in hospitalized patients. JAMA Intern Med.

[58] Gupta A (2022). Overdiagnosis of urinary tract infection linked to overdiagnosis of pneumonia: a multihospital cohort study. BMJ Qual Saf.

[59] Weinstein MP (2003). Blood culture contamination: persisting problems and partial progress. J Clin Microbiol.

[60] Hall KK, Lyman JA (2006). Updated review of blood culture contamination. Clin Microbiol Rev.

[61] Weinstein MP (1997). The clinical significance of positive blood cultures in the 1990s: a prospective comprehensive evaluation of the microbiology, epidemiology, and outcome of bacteremia and fungemia in adults. Clin Infect Dis.

[62] Becker K (2014). Coagulase-negative staphylococci. Clin Microbiol Rev.

[63] Frank KL (2008). From clinical microbiology to infection pathogenesis: how daring to be different works for Staphylococcus lugdunensis. Clin Microbiol Rev.

[64] Natsis NE, Cohen PR (2018). Coagulase-negative staphylococcus skin and soft tissue infections. Am J Clin Dermatol.

[65] Parthasarathy S (2020). Staphylococcus lugdunensis: review of epidemiology, complications, and treatment. Cureus.

[66] Leal SM (2016). Clinical significance of commensal gram-positive rods routinely isolated from patient samples. J Clin Microbiol.

[67] (2022). Global mortality associated with 33 bacterial pathogens in 2019: a systematic analysis for the Global Burden of Disease Study 2019. Lancet.

[68] Chanderraj R (2022). In critically ill patients, anti-anaerobic antibiotics increase risk of adverse clinical outcomes. Eur Respir J.

[69] Chanderraj R (2024). Mortality of patients with sepsis administered piperacillin-tazobactam vs cefepime. JAMA Intern Med.

[71] Romaschin AD (2012). Bench-to-bedside review: Clinical experience with the endotoxin activity assay. Crit Care.

[72] Shoji H (2003). Extracorporeal endotoxin removal for the treatment of sepsis: endotoxin adsorption cartridge (Toraymyxin). Ther Apher Dial.

[73] Dickson RP (2018). The lung microbiota of healthy mice are highly variable, cluster by environment, and reflect variation in baseline lung innate immunity. Am J Respir Crit Care Med.

