# Supplementary material for: Pathogen characteristics are key determinants of distinct host response phenotypes of sepsis
Source: J Clin Invest. 2026 Mar 16;136(6):e197346. doi: 10.1172/JCI197346 (PMC12987626; doi:10.1172/JCI197346)
Supplement: Supplemental data [file jci-136-197346-s196.pdf]

Supplemental material for “Pathogen characteristics are key determinants of distinct host response phenotypes of sepsis”

Authors: Rishi Chanderraj MD MSc[1,2,3], Brian Bartek MSc[4], Kathleen A. Stringer Pharm D[3,5,6], Mohamad H. Tiba MD MSc[3,7], Michael W. Sjoding MD MSc[3,5,8,9], Ying He PhD[5], Mark Nuppnau MSc[5], Kale S. Bongers MD PhD[5,10], Mark D. Adame[5,11], Sunny S. Lou MD PhD[4,12], Eric Kerschberger MD[14], Matthew M. Churpek MD, PhD, MPH [15,16], Carolyn S. Calfee MD MS[17-19], Sandhya Tripathi PhD[4], Debra M. Foster MD[20], John A. Kellum MD, MCCM[20,21], Robert P. Dickson MD[3,5,11*], Pratik Sinha, MBChB, PhD[4*]

Supplemental Methods

Clinical classifier model development

We used a previously trained and validated classifier model that utilizes the vital signs and laboratory values as inputs. The model training and validation are described in the original studies[1,2]. Briefly, hyperparameters were tuned to maximize concordance with the original LCA classification, characterized by the area under the receiver operating characteristic curve (AUROC). Model performance was maximized with a slow learning rate (η=0.01) and 2,000 learning rounds. Trees were allowed a maximum depth of two splits to reduce overfitting. XGBoost defaults were retained for γ (minimum error loss for further partitioning), λ (L2 regularization), α (L1 regularization). The final model had an AUROC=0.97 in the training data and AUROC=0.95 on an external test set, indicating high concordance with LCA-assigned phenotypes.

Infectious source adjudication

Infectious diagnoses were classified into broad categories based on prior work[3,4]We limited our analyses to patients with E. coli and S. aureus bacteremia who had a microbiologic concordance between the culture taken from the anatomic source and the organism in the bloodstream.

1. Skin, soft tissue, bone, and joint infections
2. Intrabdominal, obstetric, and gynecologic infections
3. Central nervous system infection
4. Head, neck, and odontogenic infection
5. Urinary tract infections
6. Lower respiratory tract infections
7. Bacteremia and endovascular infection

We analyzed documentation in the electronic medical record using the University of Michigan's Electronic Medical Record Search Engine (EMERSE)[5]. We used all available clinical documentation from the admission of interest.

An infectious source was assigned if 2 of 4 criteria confirmed the presence of the source.

1. An International Classification of Disease, Clinical Modification (ICD) Codes from the Ninth and Tenth revision associated with a bacterial infection based on previously validated classification schemes[3,4,6] entered into the medical record within 24 hours of initial presentation to the Emergency Department.
2. Growth of a pathogen in a sterile site culture
3. Clinical impression source of infection on admission history and physical
4. Cross-sectional imaging (CT or MRI) with radiology reports documenting the presence of infection.

Supplemental Tables

Supplemental Table 1: Comorbidities and outcomes among bacteremic patients

Characteristic	Hyperinflammatory (n=640)	Hypoinflammatory (n=1,468)	P value
Age, mean (SD)	60.78 (15.46)	60.46 (16.28)	0.672
Female N (%)	267 (41.7)	614 (41.8)	1.000
APACHE IV, mean (SD)	87.35 (29.01)	55.16 (20.93)	<0.001
Charlson index, mean (SD)	3.53 (3.13)	2.47 (2.68)	<0.001
**Comorbidities, N (%)**			
Coronary artery disease	108 (16.9%)	210 (14.3%)	0.143
Congestive heart failure	186 (29.2%)	390 (26.6%)	0.249
Peripheral artery disease	75 (11.8%)	192 (13.1%)	0.436
History of stroke	53 (8.3%)	162 (11.1%)	0.067
Dementia	26 (4.1%)	65 (4.4%)	0.799
Hemiplegia	14 (2.2%)	89 (6.1%)	<0.001
Chronic obstructive lung disease	130 (20.4%)	348 (23.7%)	0.102
Connective tissue disorder	27 (4.2%)	64 (4.4%)	0.982
Peptic ulcer disease	24 (3.8%)	37 (2.5%)	0.157
Diabetes	189 (29.6%)	445 (30.4%)	0.776
End-stage renal disease	212 (33.2%)	353 (24.1%)	<0.001
Cirrhosis	106 (16.6%)	72 (4.9%)	<0.001
Malignancy	128 (20.1%)	177 (12.1%)	<0.001
Metastatic malignancy	61 (9.6%)	91 (6.2%)	0.008
HIV	1 (0.2%)	7 (0.5%)	0.476
**Outcomes**			
90-day mortality N (%)	261 (40.8)	300 (20.4)	<0.001
Vasopressor-free days, mean (SD)	17.88 (11.94)	25.14 (7.36)	<0.001

Supplemental Table 2: Predictors of 28-day mortality among critically ill patients with sepsis (N=8,280)

Variable	HR (95% CI)	p value
Hyperinflammatory phenotype	1.04 (1.01 – 1.06)	0.0009
Age (decade)	1.01 (1.00 – 1.01)	0.003
Charlson index	1.01 (1.01 – 1.01)	< 0.001
APACHE IV	1.02 (1.02 – 1.02)	< 0.001
Male gender	1.00 (0.99 – 1.02)	0.88

Supplemental Table 3: Predictors of mortality among critically ill patients with sepsis using a continuous probability of hyperinflammatory phenotype (N=8,280)

Variable	28-day Mortality OR (95% CI)	90-day Mortality OR (95% CI)
Probability of hyperinflammatory phenotype[1]	1.05 (1.02–1.08)	1.16 (1.12–1.21)
Age (per decade)	1.01 (1.00–1.01)	1.06 (1.05–1.06)
Charlson comorbidity index	1.01 (1.01–1.01)	1.03 (1.03–1.03)
APACHE IV (per point)	1.02 (1.02–1.02)	1.00 (1.00–1.01)
Male gender	1.00 (0.99–1.02)	0.99 (0.97–1.01)
Racial minority	0.99 (0.97–1.01)	0.99 (0.96–1.01)
Time to antibiotic (per hour)	1.00 (1.00–1.00)	1.00 (1.00–1.00)

Supplemental Table 4: Lactate clearance interaction with phenotype in multivariable Cox-proportional hazards model of 90-day mortality

Characteristic	Hazard ratio (95% CI)
Hyperinflammatory subphenotype	2.21 (0.01 – 4.54)
APACHE IV (10 points)	1.20 (1.14 - 1.25)
Age (decade)	1.07 (1.03 - 1.10)
Time to antibiotic administration	1.02 (1.01 - 1.03)
Lactate clearance	1.77 (0.96 - 4.28)
Lactate clearance * Hyperinflammatory phenotype	0.42 (0.05 - 0.75)

Supplemental Table 5: Re-analysis Using 20% Lactate Reduction (ANDROMEDA-Style Threshold)

Variable	HR (95% CI)	p value
Hyperinflammatory phenotype	1.04 (1.01 – 1.06)	0.0009
Age (decade)	1.01 (1.00 – 1.01)	0.003
Charlson index	1.01 (1.01 – 1.01)	< 0.001
APACHE IV	1.02 (1.02 – 1.02)	< 0.001
Male gender	1.00 (0.99 – 1.02)	0.88

Supplemental Table 6: Distribution of detected pathogens*, corresponding hyperinflammatory probabilities

Organism	n	Mean probability	Median probability	IQR probability
Staphylococcus aureus	623	0.310	0.123	0.617
Escherichia coli	429	0.484	0.484	0.732
Klebsiella pneumoniae	206	0.419	0.328	0.689
Enterococcus faecalis	112	0.270	0.099	0.426
Enterobacter cloacae complex	108	0.527	0.577	0.690
Proteus mirabilis	82	0.486	0.484	0.749
Pseudomonas aeruginosa	82	0.480	0.492	0.830
Streptococcus anginosus group	64	0.384	0.353	0.702
Aerobic gram positive rod	51	0.240	0.074	0.403
Salmonella species	49	0.692	0.833	0.525
Streptococcus pneumoniae	46	0.431	0.271	0.747
Gram negative rod	45	0.355	0.233	0.611
Streptococcus Group B (Streptococcus agalactiae)	44	0.279	0.127	0.534
Klebsiella oxytoca	38	0.440	0.344	0.722
Anaerobic gram positive rod	38	0.395	0.181	0.739
Anaerobic gram positive cocci	37	0.203	0.100	0.222
Anaerobic non-spore forming gram positive rod	34	0.323	0.196	0.425
Bacteroides fragilis	31	0.389	0.278	0.710
Listeria species	30	0.187	0.150	0.068
Bacteroides thetaiotaomicron	29	0.504	0.428	0.619
Listeria monocytogenes	28	0.146	0.132	0.111
Pasteurella multocida	27	0.368	0.205	0.663
Lactobacillus species	27	0.087	0.038	0.069
Haemophilus influenzae	26	0.427	0.377	0.623
Citrobacter freundii complex	26	0.382	0.237	0.562
Citrobacter species	25	0.467	0.456	0.485
Gram positive cocci	25	0.060	0.049	0.081
Morganella morganii	23	0.484	0.511	0.676
Klebsiella (Enterobacter) aerogenes	23	0.444	0.353	0.734

Acinetobacter species	23	0.416	0.205	0.874
Providencia stuartii	21	0.430	0.406	0.594
Rothia mucilaginosa	20	0.459	0.445	0.634
Burkholderia cepacia complex	19	0.406	0.137	0.870
Bacteroides vulgatus	18	0.117	0.034	0.079
Klebsiella variicola	17	0.388	0.215	0.576
Actinomyces species	17	0.357	0.290	0.602
Enterococcus faecium	16	0.368	0.389	0.278
Bacillus cereus	16	0.280	0.086	0.386
Streptococcus salivarius group	16	0.218	0.091	0.236
Acinetobacter baumannii complex	14	0.520	0.555	0.499
Staphylococcus lugdunensis	14	0.314	0.187	0.393
Streptococcus bovis group	14	0.217	0.133	0.203
Cutibacterium (Propionibacterium) acnes	13	0.173	0.054	0.036
Bacteroides caccae	12	0.639	0.806	0.312
Corynebacterium striatum	12	0.592	0.624	0.689
Clostridium species, not perfringens or septicum	12	0.478	0.614	0.628
Anaerobic gram negative cocci	12	0.414	0.376	0.056
Aerococcus species	12	0.132	0.050	0.120
Bacteroides species	11	0.541	0.601	0.310
Haemophilus parainfluenzae	11	0.269	0.121	0.369
Prevotella buccae	11	0.255	0.177	0.151
Parvimonas micra	11	0.125	0.050	0.035
Anaerococcus vaginalis	11	0.109	0.041	0.094
Aerococcus viridans	10	0.580	0.839	0.673
Vancomycin resistant Enterococcus faecium	10	0.202	0.280	0.309
Alcaligenes faecalis	10	0.146	0.001	0.148
Acremonium species	9	0.623	0.760	0.200
Bacteroides ovatus	9	0.616	0.722	0.306
Fusobacterium species	9	0.470	0.674	0.793
Cardiobacterium species	9	0.197	0.029	0.222
Rothia dentocariosa	9	0.169	0.050	0.130
Serratia marcescens	8	0.576	0.809	0.801
Actinomyces species, not israelii	8	0.297	0.089	0.434

Clostridium tertium	7	0.551	0.752	0.633
Clostridium perfringens	7	0.453	0.451	0.479
Clostridium septicum	7	0.442	0.446	0.554
Aeromonas species	7	0.407	0.025	0.901
Parabacteroides distasonis	7	0.359	0.192	0.591
Neisseria meningitidis	7	0.335	0.303	0.280
Herbaspirillum species	7	0.099	0.108	0.051
Actinomyces neuii	6	0.371	0.253	0.012
Campylobacter fetus	6	0.295	0.050	0.536
Vibrio species	6	0.282	0.148	0.259
Neisseria species	6	0.260	0.179	0.330
Streptococcus mutans group	6	0.250	0.333	0.312
Corynebacterium minutissimum	6	0.064	0.027	0.028
Capnocytophaga species	4	0.653	0.859	0.215
Clostridium clostridiiforme complex	4	0.428	0.411	0.773
Facklamia languida	4	0.381	0.262	0.419
Streptococcus pneumoniae - Mucoid variety	4	0.365	0.365	0.537
Paenibacillus species	4	0.352	0.421	0.336
Citrobacter koseri	4	0.347	0.347	0.608
Fusobacterium necrophorum	4	0.318	0.174	0.286
Vancomycin resistant Enterococcus faecalis	4	0.283	0.283	0.526
Gemella haemolysans	4	0.250	0.274	0.262
Aggregatibacter aphrophilus	4	0.245	0.216	0.403
Clostridium sordellii	4	0.234	0.144	0.216
Pantoea species	4	0.215	0.039	0.215
Granulicatella adiacens	4	0.168	0.062	0.171
Clostridium butyricum	4	0.166	0.095	0.123
Campylobacter ureolyticus	4	0.155	0.000	0.155
Nocardia nova	4	0.126	0.129	0.041
Eggerthella lenta	4	0.110	0.124	0.070
Corynebacterium species	4	0.099	0.080	0.075
Clostridium paraputrificum	4	0.061	0.058	0.046
Corynebacterium jeikeium (Group JK)	4	0.017	0.013	0.027
Clostridium innocuum	3	0.526	0.675	0.275
Escherichia vulneris	3	0.518	0.739	0.332

Pseudomonas mendocina	3	0.517	0.337	0.270
Clostridium ramosum	3	0.456	0.455	0.445
Clostridium spp	3	0.411	0.416	0.343
Gemella morbillorum	3	0.396	0.469	0.359
Enterobacter species	3	0.384	0.145	0.358
Dialister pneumosintes	3	0.209	0.259	0.184
Bacteroides fragilis group	3	0.013	0.018	0.011
Proteus vulgaris	2	0.208	0.208	0.000
Pseudomonas aeruginosa (mucoid variety)	2	0.067	0.067	0.000
Cronobacter species	2	0.049	0.049	0.022
Streptococcus mutans	2	0.005	0.005	0.000
Gram negative rod, not Bacteroides fragilis group	1	0.079	0.079	0.000
Fusobacterium nucleatum	1	0.070	0.070	0.000
Klebsiella species	1	0.002	0.002	0.000
Dermabacter hominis	1	0.000	0.000	0.000

***Organisms are listed at the individual isolate level. The analytic cohort included 2,108 unique bacteremia episodes, but the total organism count is higher owing to polymicrobial infections, in which multiple species were isolated from a single episode.**

Supplemental Table 7: Probability of hyperinflammatory phenotype among patients with lower respiratory tract infection and without bacteremia by causative pathogen

Source	Genus (combined)	n cases	Mean P(hyper)	Median P(hyper)	IQR (25–75%)
LRI	*Enterobacterales*	115	0.289	0.101	0.017 – 0.520
	Staphylococcus	204	0.25	0.09	0.017 – 0.368
	Streptococcus	35	0.245	0.097	0.036 – 0.453
	Pseudomonas	60	0.166	0.021	0.005 – 0.148
UTI	*Enterobacterales*	369	0.297	0.118	0.016 – 0.578
	Pseudomonas	31	0.215	0.066	0.011 – 0.354
	Enterococcus	63	0.159	0.046	0.013 – 0.329
	Streptococcus	14	0.121	0.066	0.012 – 0.171

Supplemental Table 8: Lactate clearance interaction with Enterobacterales bacteremia in multivariable Cox-proportional hazards model of 90-day mortality

Characteristic	Hazard ratio (95% CI)
Enterobacterales	1.25 (0.12 – 2.54)
APACHE IV (10 points)	1.21 (1.12 - 1.27)
Age (decade)	1.07 (1.03 - 1.10)
Time to antibiotic administration	1.12 (1.01 - 1.23)
Lactate clearance	0.57 (0.06 – 0.97)
Lactate clearance * Enterobacterales	0.25 (0.01 - 0.54)

Supplemental Table 9: Distribution of Hyperinflammatory Probabilities by Infection Source for *Staphylococcus aureus*

Infection Source (Group)	n cases	Mean Probability	Median Probability	IQR (25 – 75 %)
Pneumonia	25	0.63	0.63	0.53 – 0.75
Skin / Soft Tissue	39	0.38	0.37	0.26 – 0.54
Prosthetic / Line-related	92	0.39	0.37	0.27 – 0.51
Other Sites	69	0.34	0.3	0.19 – 0.45

Supplemental Table 10: Distribution of Hyperinflammatory Phenotype Probabilities by Infection Source for *E. coli*

Infection Source (Group)	n cases	Mean Probability	Median Probability	IQR (25 – 75 %)
Abdominal infection	15	0.9	0.88	0.82 – 0.99
Pneumonia	53	0.54	0.55	0.23 – 0.79
Urinary tract infection	57	0.48	0.47	0.30 – 0.62
Other sites	30	0.32	0.28	0.17 – 0.44

Supplemental Figures

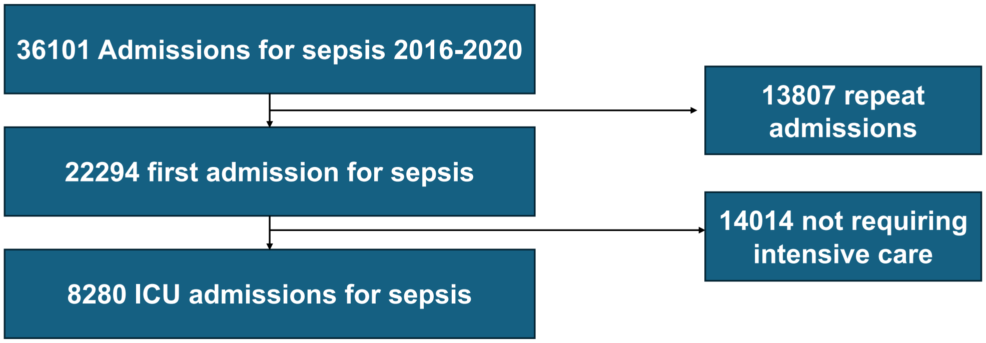

Supplemental Figure 01: Cohort flow diagram.

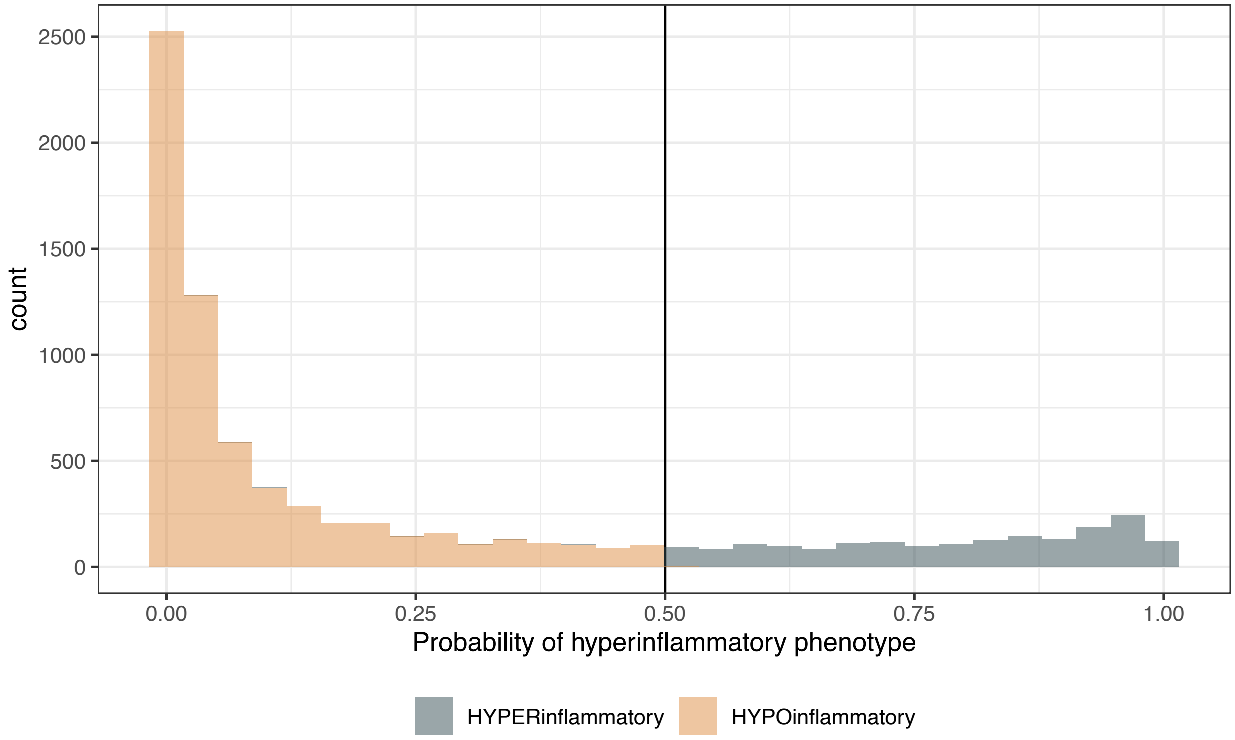

Supplemental Figure 2: Histogram of patients by probability of belonging to the hyperinflammatory subphenotype.

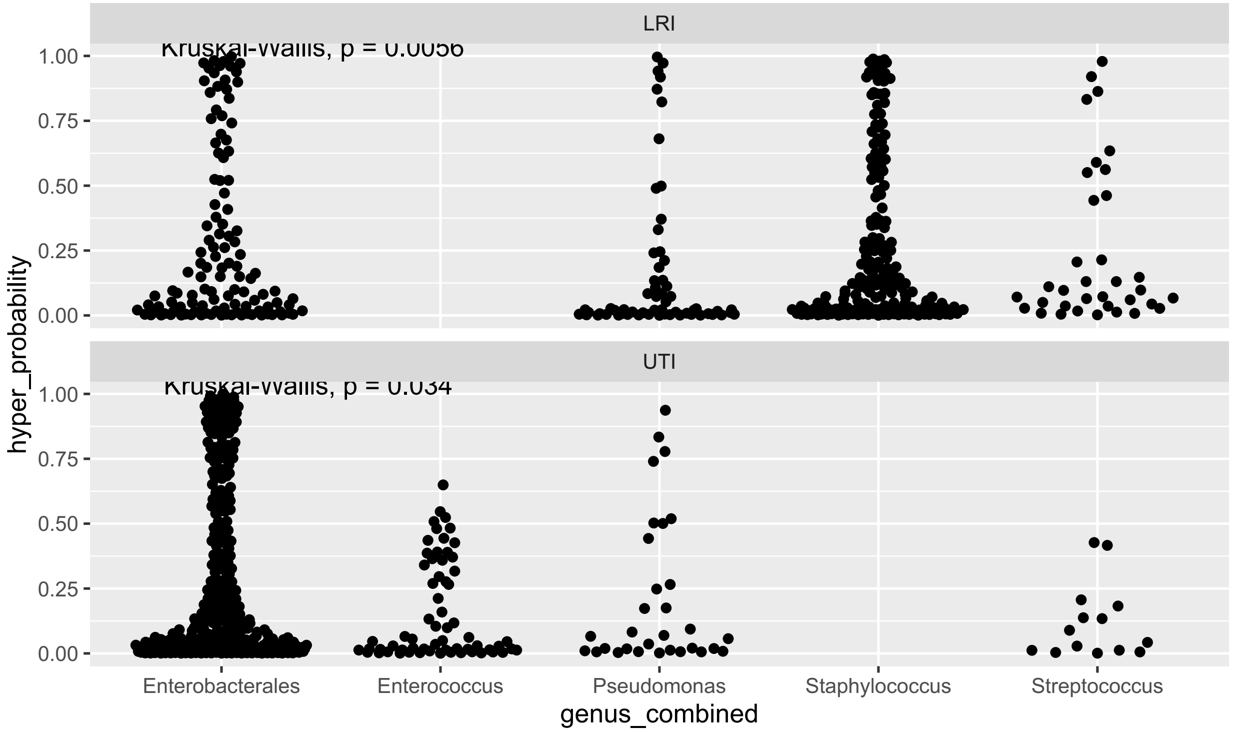

Supplemental Figure 3: Enterobacterales infections showed the highest hyperinflammatory probabilities in patients without bacteremia in lower respiratory tract infections (*p = 0.0056*) and UTI (*p = 0.034*, Kruskal–Wallis)

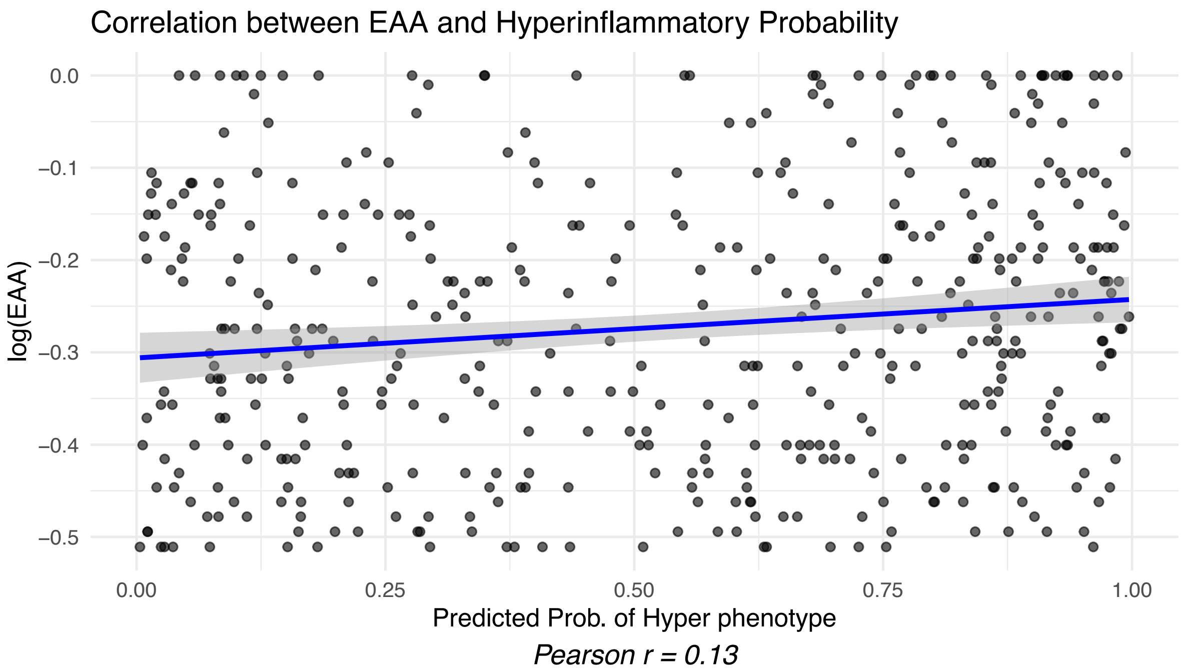

Supplemental Figure 4: Correlation between endotoxin and hyperinflammatory phenotype probability. We observed a significant positive correlation between EAA and hyperinflammatory probability (Spearman ρ = 0.13, *p = 0.003* overall; ρ = 0.21, *p = 0.001* within the hyperinflammatory subset)

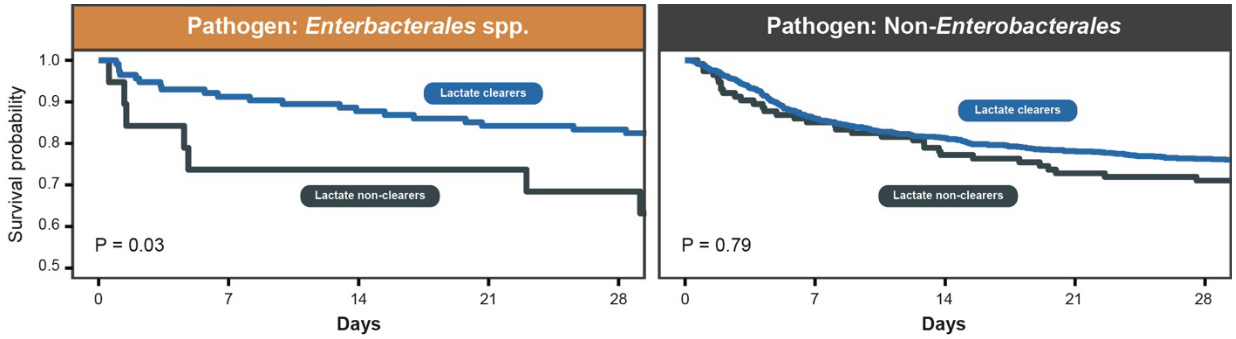

Supplemental Figure 5: When comparing 28-day mortality, initial lactate clearance (defined as decreasing initial lactate concentration by 10% within 2-12 hours) predicted mortality in patients with *Enterobacterales* spp. infections, but not in patients who did have *Enterobacterales* spp. infections.

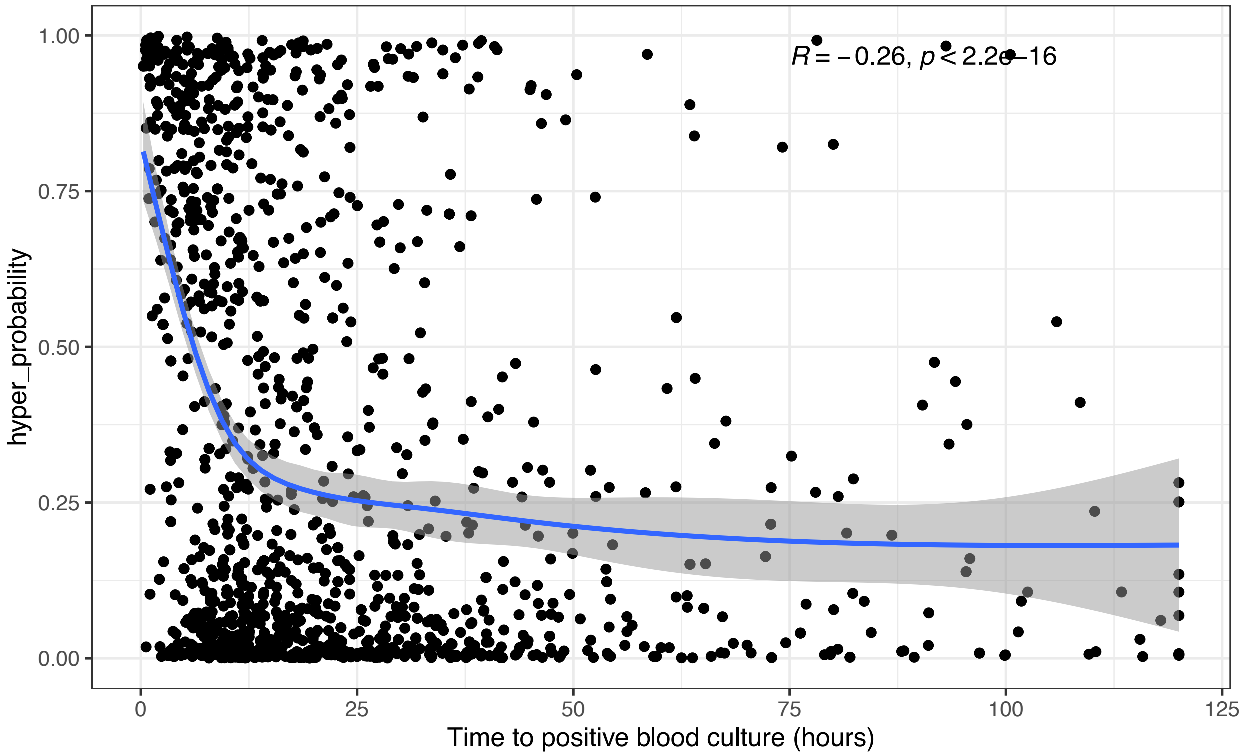

Supplemental Figure 6: Relationship between time to positive blood culture and probability of hyperinflammatory subphenotype.

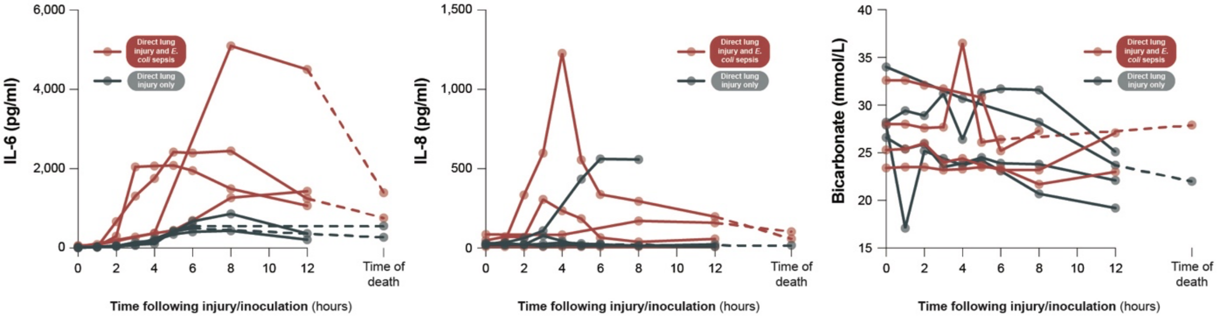

Supplemental Figure 7: Individual biomarker trajectories for all experimental pigs.

Supplementary References

1. Sinha, P.*, et al.* Identifying molecular phenotypes in sepsis: an analysis of two prospective observational cohorts and secondary analysis of two randomised controlled trials. *Lancet Respir Med* **11**, 965-974 (2023).
2. Sinha, P.*, et al.* Development and validation of parsimonious algorithms to classify acute respiratory distress syndrome phenotypes: a secondary analysis of randomised controlled trials. *Lancet Respir Med* **8**, 247-257 (2020).
3. Rhee, C.*, et al.* Prevalence of Antibiotic-Resistant Pathogens in Culture-Proven Sepsis and Outcomes Associated With Inadequate and Broad-Spectrum Empiric Antibiotic Use. *JAMA Netw Open* **3**, e202899 (2020).
4. Tedijanto, C., Nevers, M., Samore, M.H. & Lipsitch, M. Antibiotic Use and Presumptive Pathogens in the Veterans Affairs Healthcare System. *Clin Infect Dis* **74**, 105-112 (2022).
5. Hanauer, D.A., Mei, Q., Law, J., Khanna, R. & Zheng, K. Supporting information retrieval from electronic health records: A report of University of Michigan's nine-year experience in developing and using the Electronic Medical Record Search Engine (EMERSE). *J Biomed Inform* **55**, 290-300 (2015).
6. Chua, K.P., Fischer, M.A. & Linder, J.A. Appropriateness of outpatient antibiotic prescribing among privately insured US patients: ICD-10-CM based cross sectional study. *BMJ* **364**, k5092 (2019).
